# The Facial Action Coding System for Characterization of Human Affective Response to Consumer Product-Based Stimuli: A Systematic Review

**DOI:** 10.3389/fpsyg.2020.00920

**Published:** 2020-05-26

**Authors:** Elizabeth A. Clark, J'Nai Kessinger, Susan E. Duncan, Martha Ann Bell, Jacob Lahne, Daniel L. Gallagher, Sean F. O'Keefe

**Affiliations:** Virginia Polytechnic Institute and State University, Blacksburg, VA, United States

**Keywords:** emotions, facial action coding system, action units, facial analysis, consumers

## Abstract

To characterize human emotions, researchers have increasingly utilized Automatic Facial Expression Analysis (AFEA), which automates the Facial Action Coding System (FACS) and translates the facial muscular positioning into the basic universal emotions. There is broad interest in the application of FACS for assessing consumer expressions as an indication of emotions to consumer product-stimuli. However, the translation of FACS to characterization of emotions is elusive in the literature. The aim of this systematic review is to give an overview of how FACS has been used to investigate human emotional behavior to consumer product-based stimuli. The search was limited to studies published in English after 1978, conducted on humans, using FACS or its action units to investigate affect, where emotional response is elicited by consumer product-based stimuli evoking at least one of the five senses. The search resulted in an initial total of 1,935 records, of which 55 studies were extracted and categorized based on the outcomes of interest including (i) method of FACS implementation; (ii) purpose of study; (iii) consumer product-based stimuli used; and (iv) measures of affect validation. Most studies implemented FACS manually (73%) to develop products and/or software (20%) and used consumer product-based stimuli that had known and/or defined capacity to evoke a particular affective response, such as films and/or movie clips (20%); minimal attention was paid to consumer products with low levels of emotional competence or with unknown affective impact. The vast majority of studies (53%) did not validate FACS-determined affect and, of the validation measures that were used, most tended to be discontinuous in nature and only captured affect as it holistically related to an experience. This review illuminated some inconsistencies in how FACS is carried out as well as how emotional response is inferred from facial muscle activation. This may prompt researchers to consider measuring the total consumer experience by employing a variety of methodologies in addition to FACS and its emotion-based interpretation guide. Such strategies may better conceptualize consumers' experience with products of low, unknown, and/or undefined capacity to evoke an affective response such as product prototypes, line extensions, etc.

## Introduction

A variety of methods exist to capture the human affectual experience in response to stimuli; however, the nature of emotions makes them difficult to measure and interpret. In recent years, there has been an expanding body of research investigating how specific affectual responses (i.e., emotional response) can systematically influence an individual's perceptions, judgments, and behaviors to products and environment (Lerner and Keltner, 2000, 2001; Lewis, 2000; Tiedens and Linton, 2001; Garg et al., 2005; Maheswaran and Chen, 2006; Agrawal et al., 2007, 2013; Han et al., 2007; de Hooge et al., 2008; Keltner and Lerner, 2010). Emotions are short-term, complex, multidimensional behavioral responses (Smith and Ellsworth, 1985; Lambie and Marcel, 2002) that are aimed to promote adaptive strategies in a variety of contexts. Specific emotions are functions of a multitude of information-rich associations underlying the emotional experience (So et al., 2015). Emotional appraisals (i.e., how these specific emotions are elicited and function to influence an individual's conscious and unconscious evaluations of events and situations) have been widely recognized to impact decision-making and other cognitive processes (Scherer, 2001; Tiedens and Linton, 2001; Herrald and Tomaka, 2002; Maheswaran and Chen, 2006; Raghunathan et al., 2006; de Mello et al., 2007; Kim et al., 2010; Hill et al., 2011; Wilcox et al., 2011; Winterich and Haws, 2011; Mirabella, 2014).

Though there is no consensus on a definition for emotions, the range of effects that emotions have on facial expression is recognized as broad and diverse (Jack et al., 2012). Emotions have been traditionally characterized dichotomously as either positive (e.g., joyful, content, passionate) or negative (e.g., disgust, worried, anxious) affective states (Plutchik, 1980; Russell, 1980). Positive affect refers to the extent an individual feels enthusiastic, active, and pleasurably aroused whereas negative affect refers to the extent an individual feels upset, distressed, and unpleasantly aroused (Watson and Tellegen, 1985). However, Lazarus (Novacek and Lazarus, 1990; Lazarus, 1991a,b) proposed a theory in which motivational processes played a central role in emotional expression. In this theory, emotions such as pride, love, or happiness would arise when a situation is regarded as beneficial and anger, anxiety, and sadness would arise when a situation is regarded as harmful. Davidson (1984) proposed a similar model linked to frontal electroencephalogram (EEG) asymmetry in the brain during emotional states. He proposed that frontal asymmetry was not related to the valence of emotional stimulus but rather to the motivational system that is engaged by the stimulus. He concluded that emotion will be associated with a right or left asymmetry depending on the extent to which it is accompanied by approach or withdrawal behaviors (Davidson, 1984). Recent work has supported distinguishing facially expressed emotions as approach or withdrawal based on the relationship between emotions and cognitive processes (Coan et al., 2001; Alves et al., 2009; van Peer et al., 2010).

Tomkins (1992) defined the subjective experience of emotion as the feedback from facial muscular changes, and research by others has investigated how an individual's subjective experience of emotions influences performance of different muscular movements (Laird, 1974; Izard, 1977; Tourangeau and Ellsworth, 1979). From this research, Ekman et al. (1972) hypothesized that the face may also influence other people's emotional experiences by providing signals about how an individual feels. In an effort to provide a sounder basis about what different facial actions might signify, Ekman and Friesen (1976, 1978) developed a novel technique for measuring facial behavior, the Facial Action Coding System (FACS). FACS was primarily developed as a comprehensive system to distinguish all possible visible anatomically based facial movements (Ekman and Friesen, 1976). FACS provides a common nomenclature for facial movement research, which allows for diverse application in a variety of fields. Following the work of Hjortsjö (1970); Ekman and Friesen (1976, 1978) identified and published a system of action units (AU) or fundamental actions of individual or group muscles. AUs are identified by a number, shorthand name, and include the anatomical basis for each action (Table 1) and are rated on a 5-point intensity scale (A = trace, B = slight, C = marked or pronounced, D = severe or extreme, E = maximum). Ekman et al. (2002) later published a significant update to FACS. Although FACS itself includes no emotion-specified descriptors, it is commonly used to interpret non-verbal communicative signals, such as facial expressions related to emotion or other human states (Valstar and Pantic, 2006); related resources such as EMFACS (emotional FACS), the FACS Investigators' Guide (Ekman et al., 2002), as well as the FACS interpretive database (Ekman et al., 1998) are used to make emotion-based inferences from single and/or combinations of AUs. A constraint of FACS is that it deals with *clearly* visible changes in facial movement and doesn't account for subtle visible changes such as changes in muscle tonus (Ekman and Friesen, 1976). Another limitation of FACS is that it was developed to measure movement in the face, thus other facial phenomena (e.g., changes in skin coloration, sweating, tears, etc.) are excluded.

**Table 1:** Single action units in the facial action code Ekman et al., 2002.

**AU number**	**Name of action**	**Muscle(s) activated**
1	Inner brow raiser	Frontalis (pars medialis)
2	Outer brow raiser	Frontalis (pars lateralis)
4	Brow lowerer	Depressor glabellae, depressor supercilii, corrugator supercilli
5	Upper lid raiser	Levator palpebrae superioris, superior tarsal muscle
6	Cheek raiser	Orbicularis oculi (pars orbitalis)
7	Lid tightener	Orbicularis oculi (pars palpebralis)
8	Lips toward each other	Orbicularis oris
9	Nose wrinkler	Levator labii superioris alaeque nasi
10	Upper lid raiser	Levator labii superioris, caput infraorbitalis
11	Nasolabial deepener	Zygomaticus minor
12	Lip corner puller	Zygomaticus major
13	Sharp lip puller	Levator anguli oris (i.e., caninus)
14	Dimpler	Buccinnator
15	Lip corner depressor	Depressor anguli oris (i.e., triangularis)
16	Lower lip depressor	Depressor labii inferioris
17	Chin raiser	Mentalis
18	Lip pucker	Incisivii labii superioris and incisivii labii inferioris
19	Tongue Show	
20	Lip stretcher	Risorius with platysma
21	Neck tightener	Platysma
22	Lip funneler	Orbicularis oris
23	Lip tightener	Orbicularis oris
24	Lip pressor	Orbicularis oris
25	Lips part	Depressor labii inferioris or relaxation of mentalis, or orbicularis oris
26	Jaw drop	Masetter; relaxed temporalis and internal pterygoid
27	Mouth stretch	Pterygoids, digastric
28	Lip suck	Orbicularis oris
41	Lid droop	Relaxation of levator palpebrae superioris
42	Slit	Orbicularis oculi (pars palpebralis)
43	Eyes closed	Relaxation of levator palpebrae superioris, orbicularis oculi (pars palpebralis)
44	Squint	Orbicularis oculi (pars palpebralis)
45	Blink	Relaxation of levator palpebrae superioris, orbicularis oculi (pars palpebralis)
46	Wink	Relaxation of levator palpebrae superioris, orbicularis oculi (pars palpebralis)

*The coded numbers are arbitrary and do not correspond to any significant value*.

Although FACS was designed for manual application by trained and certified FACS coders, the subjectivity and time intensiveness of human-performed coding had led to the adoption of FACS into computer automated systems (Hamm et al., 2011). Automated facial expression analysis (AFEA) was developed to reduce the challenges of manual FACS application. AFEA provides more rapid evaluation of facial expressions and subsequent classification of those expressions into discrete categories of basic emotions (happy, sad, disgusted, surprised, angry, scared, and neutral) on a scale from 0 (not expressed) to 1 (fully expressed) (Lewinski et al., 2014d). Additionally, software may assess AU activation, intensity, and duration. Several commercially available software systems can generate AFEA. As an example, the integration of FACS and emotional characterization approach for one software system (Noldus FaceReader™; http://www.noldus.com; Noldus Information Technology, 2014) is described. The face reading software functions by finding a person's face and subsequently creating a 3D Active Appearance Model (AAM) (Cootes and Taylor, 2000) of the face. The AAM serves as a base for which all the universal emotional expressions, plus the neutral expression, can be identified via independent neural network classifiers trained with backpropagation—a method proven effective for pattern recognition tasks (Bishop, 1995). The final expression judgement of a face image is then based on the network with the highest output. The automatic emotions expression classifiers used in Noldus FaceReader™ were trained on the “Karolinska Directed Emotional Faces” set containing 980 high quality facial images showing one of the universal emotional expressions or a neutral expression; 89% of all faces presented to the classifier is classified correctly which, at the time of its development, was promising as it was among the highest reported results on emotional expression classification from static images (van Kuilenburg et al., 2005). Similarly, independent classifiers, which perform FACS scoring of a face, were trained on 858 appearance vectors of images from the Cohn-Kanade AU-Coded Facial Expression Database; AUs were detected with an average accuracy of 86%, which means that most classified faces will have one or more AUs scored incorrectly. More recently, Noldus FaceReader™ (version 6.0) has proven to be a reliable indicator of facial expressions of basic emotions, although it could stand to become more robust with respect to FACS coding (Lewinski et al., 2014d).

AFEA technology has been used in a variety of consumer-affective research and marketing studies (de Wijk et al., 2012, 2014; He et al., 2012a,b, 2014, 2016, 2017; Garcia-Burgos and Zamora, 2013; Lewinski et al., 2014a,b,c; Chavaglia and Filipe, 2015; Crist et al., 2016; Mozuriene et al., 2016; Walsh et al., 2017a,b); however, these studies fail to characterize the role of FACS, specifically AUs, as it pertains to consumer emotional behavior and stimuli evaluation. Additional approaches exist, such as those from the field of neuromarketing, to understand how internal and external forces (e.g., an individual's internal emotional experience vs. emotional expression by entities outside of the individual) might shift consumers from one pattern of decisions to another (Breiter et al., 2015). For example, functional magnetic resonance imaging (fMRI) is employed to map differences in brain region activity and, subsequently, to infer systematic variations in the engagement of emotions (Poldrack, 2006, 2008). Likewise, facial electromyography (EMG) serves to measure and map the underlying electrical activity that is generated when muscles contract during discrete choice-making (Rasch et al., 2015) while eye-tracking technology has been harnessed to study gaze behavior with respect to packaging, label and menu design, in-store consumer behavior, emotional responses, and eating disorders (Duerrschmid and Danner, 2018). In fact, neuromarketing techniques are often used in conjunction with FACS to provide a more holistic picture of the consumer affective experience (Cohn et al., 2002; Balzarotti et al., 2014; Lagast et al., 2017).

Given the aforementioned application of FACS to measure emotion in various scientific fields, the aim of this review is to understand how the facial action coding system (manual or automated) has been used to investigate human emotional behavior to consumer product-based stimuli. This review will outline the methods and purposes for using FACS to characterize human affective (i.e., emotional) responses elicited by consumer product-based stimuli and, additionally, will provide evidence for how FACS-measured affective responses are validated.

## Methods

### Study Design and Systematic Review Protocol

The search for articles was carried out between June 2018 and June 2019. The syntax was developed in line with common search strategies in consumer and sensory research (Booth, 2014) and in line with studies on emotion in the field of psychology (Mauss and Robinson, 2009). The search included an *a priori* limit for only human studies and restricted the publication year to studies published after 1978 since the defining work on the Facial Action Coding System was published in 1978 (Ekman and Friesen, 1978). The search syntax was developed from key terms within the PICOS framework (Table S2), which were combined using the Boolean operator “OR” and between elements using the Boolean operator “AND.” This resulted in the combination of the following keywords: (“facial action” OR “automatic facial expression analysis”) AND (consum^*^ OR panel^*^ OR human OR participant OR customer OR client OR purchaser OR user OR buyer OR shopper OR patron OR vendee). Peer reviewed articles that investigated (i) method of FACS implementation, (ii) purpose of study, (iii) consumer product-based stimuli used, and (iv) measures of affect validation were obtained from the databases ABI/Inform, ISI Web of Science, PsychINFO, PubMed as well as Google Scholar. To be included in the systematic review, studies had to be published in English, used the FACS system or its AUs to evaluate facial response to purchasable consumer-based goods or products (stimuli) that evoke at least one of the five senses (sight, touch, smell, taste, hearing), and reported outcomes on emotion, mood, or arousal and outcomes on measures used to validate emotions, mood, or arousal. Studies were excluded if they were not full-text articles, written in a language other than English, conducted solely on animals, measured and/or characterized affective response to media-based messaging, webpages, or forums used to market and/or sell consumer products, did not use the facial action coding system and/or the facial action units of the facial action coding system to measure and/or characterize emotion, and if they included participants with eating disorders (i.e., anorexia nervosa, bulimia nervosa), neurologic, psychiatric, or physiological disorders. Inclusion and exclusion criteria are shown in Table S1. This review focused on research studies that used FACS to measure human affective responses to consumer product-based stimuli with no limitation in setting. As this review aims to compare different methods of FACS implementation, no exclusions were made based on comparison.

### Data Sources, Studies Sections, and Data Extraction

This search syntax was used in all databases (ABI/Inform, ISI Web of Science, PsychINFO, PubMed) as well as Google Scholar and database index terms or headings were checked for unique terms to add to the search. Additionally, known systematic reviews on the facial action coding system or facial expression of emotion were considered and both their cited and citing sources were reviewed for potential inclusion in this study. Included studies' reference lists and studies citing the included studies were also reviewed for inclusion.

All papers retrieved were subsequently imported into both a citation manager library (Zotero, version 5.0.55, Center for History and New Media at George Mason University, Fairfax, VA, USA) and a systematic review management database/software (Covidence, version 1052 cd18d6a1, Melbourne, Victoria, Australia) and duplicates were removed. Two researchers conducted the search independently, in accordance with the Preferred Reporting Items for Systematic Reviews and Meta-Analyses (PRISMA) Statement (Moher et al., 2009), using the same databases and all findings were merged. If the researchers encountered conflicts in their independent assessments, they were discussed until a consensus was reached. The first step of the search was based on a title and abstract screening for existence of important key words related to the research question and for relevance of the studies based on the inclusion and exclusion criteria. In the second step, all relevant articles were subjected to an in-depth full-text critical review for eligibility.

**Figure 1:**
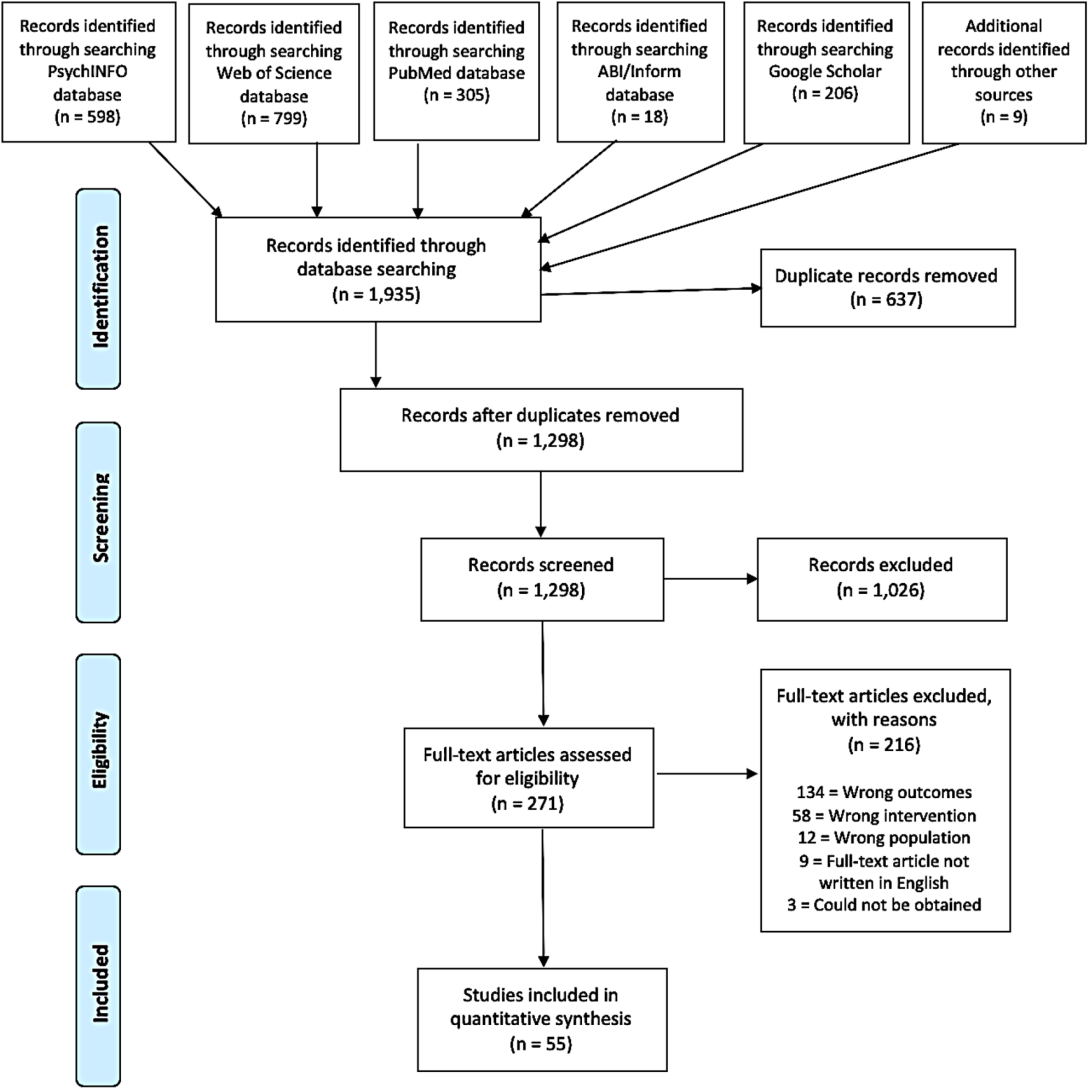
Flow diagram of literature search and selection procedure employed to identify studies eligible for inclusion in the systematic review.

The search resulted in an initial total of 1,935 records, of which 18 were found in ABI/Inform, 598 in PsychINFO, 799 in Web of Science, 305 in PubMed, and 206 in Google Scholar. Additionally, 9 studies were later included after reviewing reference lists and studies citing the included studies. A total of 638 duplicates were removed, resulting in 1,298 records to be screened. Based on title and abstract screening for existence of important keywords related to research question as well as inclusion and exclusion criteria, 1,026 were found to be irrelevant and 271 records were subject to a full-text review. After the full-text review, 216 articles were found not eligible for inclusion based on the following criteria: wrong outcomes (*n* = 134), wrong intervention (*n* = 58), full-text article not written in English (*n* = 9), wrong population (*n* = 12), full-text article not accessible (*n* = 3). A total 55 articles were selected for extraction and analysis. The search strategy for this systematic review can be found in Figure 1, which is based on the PRISMA (Moher et al., 2009).

### Data Analysis

To have a comprehensive understanding of the characteristics of the studies, a data extraction sheet was developed in Microsoft Excel (version 16.16.2., Microsoft Corporation, Redmond, WA, USA). Identifying information extracted from the studies including the title, author(s), publishing year, and Covidence number were placed into separate columns. Columns were also made for information extracted for each outcome of interest (i) method of FACS implementation (ii) purpose of study (iii) consumer product-based stimuli used and (iv) measures of affect validation; a “Notes” column was also made so further detail could be provided on each outcome of interest, if necessary (Table S3). Method of FACS implementation consisted of three broad categories: manual, automatic, and “both” (a combination of manual as well as automatic). For purpose of study, typology-based categories were developed to structure the plethora of aims represented across the extracted studies (Table S4). Likewise, typology-based categories were developed to structure the diversity of diversity of purchasable, consumer product-based stimuli that were represented across the extracted studies (Table S4). If a study utilized multiple stimuli, then the “Multiple” category was chosen, and all stimuli were listed out in the “Notes” column of Table S3. An variety of affect validation measures represented across the extracted studies (Table S4). These categories are defined by the nature of the validation measures, which included externally-reported (i.e., non-self-reported) measures (implicit: affect-related activation and/or arousal occurring within the body; affect-related activation and/or arousal occurring within the body; explicit: affect-related activation and/or arousal that can be observed occurring on the outside of the body) and self-reported measures (vocalized: vocal communication of information pertaining to arousal and/or affective state; non-vocalized: information pertaining to arousal or affective state that is physically input, selected, or expressed). The category “None” was used to describe studies where no additional measure was used to validate FACS-measured affective response. For interpretations for key words and terms used throughout the review has been provided (Table S5). If the researchers encountered conflicts in their independent assessments, they were discussed until a consensus was reached.

Additionally, all extracted studies were assessed for risk of bias using the Cochrane Risk of Bias Tool (Higgins and Green, 2011). Bias was assessed in the following categories: selection bias (allocation concealment, sequence generation), performance bias (blinding of participants and personnel to all outcomes), detection bias (blinding of assessors to all outcomes), attrition bias (incomplete outcome data), reporting bias (selective outcome reporting), and other sources of bias. The two researchers conducted the risk of bias assessment independently and evaluated the extracted studies for selection, performance, detection, attrition, reporting, and other sources of bias, which was rated as being either low, high, or unclear. If the researchers encountered conflicts in their independent assessments, they were discussed until a consensus was reached.

## Results

### Overview of the Selected Articles

**Figure 2:**
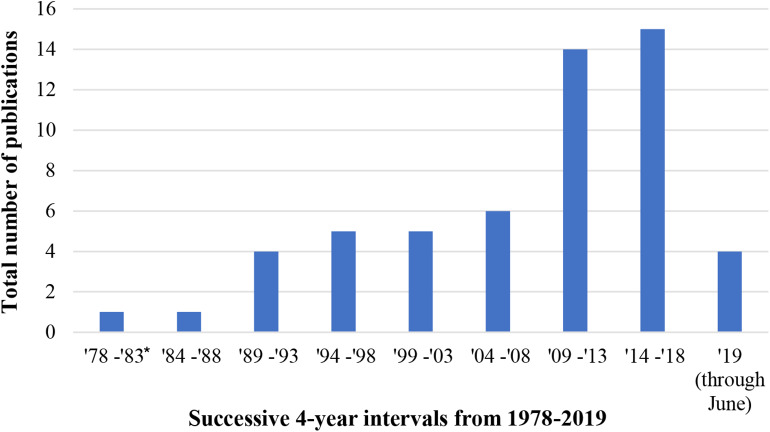
Total number of publications included in this review (*n* = 55) as published over successive 4-years intervals from 1978 to 2019. ^*^ This is a 5 year interval to capture the year FACS was established [1978]

**Figure 3:**
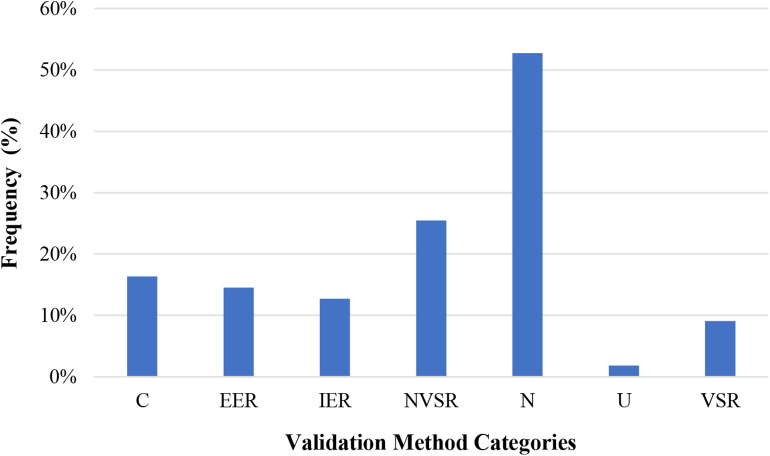
Frequency of validation methods used within 55 studies (C, Combination of methods; EER, Explicit externally-reported; IER, implicit externally-reported; NVSR, Non-vocalized self-reported; N, None or no validation measures used; U, Unsure if self-reported method was vocalized or non-vocalized; VSR, Vocalized self-reported).

Table 2 gives a summary of the final set of 55 articles with respect to their purpose, how FACS was implemented, the consumer product-based stimuli used, and measures utilized to validate FACS-determined affect (i.e., emotion). The data extraction sheet for this review, which outlines all selected article, outcomes of interest, and notes providing further detail about the outcomes has also been provided (Table S3). More than half of these articles were published in the last 10 years (33 studies; 60%) of which a majority have been published in the last 5 years (19 studies; 35%). About 13 times more articles were published in the last 4 years than during the first 4 years (Figure 2). This suggests that there is a growing interest in using FACS to measure human emotional behavior to consumer product-based stimuli. Most studies implemented FACS manually (40 studies; 73%), however studies published within the last 5 years (*n* = 19) have increasingly implemented FACS automatically (9 studies; 47%; automatic or both automatic and manual within last 5 years) to assess human affective response. Though the studies were performed for a variety of purposes, the most frequent purposes were to develop products & software (11 studies; 20%) as well as to assess behavior toward stimuli under a social and/or situational context (10 studies; 18%) and investigate human development and/or behavior (10 studies; 18%). Films and/or movie clips (11 studies; 20%) as well as comedies, jokes, or cartoons (10 studies; 18%) were used most often as the emotion-evoking consumer product-based stimuli; though several studies used multiple stimuli (9 studies; 16%). Figure 3 gives an overview of the consumer product-based stimuli that have been used to elicit an affective response. Most often, studies validated an individual's FACS-determined affective response with non-vocalized self-reported measures (12 studies; 22%) via questionnaires, scales, or surveys. However, it should be noted that the vast majority of studies (29 studies; 53%) did not use another measure to validate an individual's affective response as determined using FACS.

**Table 2:** Overview of the studies included in the systematic review by outcome of interest including the Facial Action Coding System (FACS) application, purpose of study, products assessed, and validation method in the study.

**MANUAL**						
**Purpose of study - stimuli (i.e., product) category**	**Validation method implicit externally-reported measures**	**Explicit externally-reported measures**	**Vocalized self-reported measures**	**Non-vocalized self-reported measures**	**Combination**	**None**
**Affective states during learning**						
- Tutoring program/system			Craig et al., 2008			Grafsgaard et al., 2011
**Behavior toward stimuli under social and/or situational contexts**						
- Cigarettes						Sayette et al., 2005
- Comedy, joke, and/or cartoon				Sayette et al., 2019		Ruch, 1997b; Lynch and Trivers, 2012
- Computer game						Mui et al., 2017
- Film and/or movie clip				Jakobs et al., 1999		
- Multiple						Dale et al., 1991
- Physical game						Schneider and Josephs, 1991
**Facial behavior and emotion expression**						
- Comedy, joke, and/or cartoon				Krumhuber and Manstead, 2009		Ruch, 1997a
- Film and/or movie clip				Ekman et al., 1980; Dosmukhambetova and Manstead, 2012; Namba et al., 2017	Frank et al., 1997	
- Multiple						Catia et al., 2017
- Odor				Soussignan and Schaal, 1996		
**Genetics and emotion expression**						
- Comedy, joke, and/or cartoon						Haase et al., 2015
**Human development and/or behavior**						
- Cigarettes						Sayette and Hufford, 1995; Griffin and Sayette, 2008; Sayers and Sayette, 2013
- Comedy, joke, and/or cartoon						Lynch, 2010
- Film and/or movie clip					Johnson et al., 2017	
- Food						Forestell and Mennella, 2012
- Multiple						Sayette and Parrott, 1999
- Odor					Soussignan et al., 1999	
- Physical game						Unzner and Schneider, 1990
- Toy					Cole et al., 1994	
**Human-computer interaction and emotion**						
- Computer game					Balzarotti et al., 2014	
- Film and/or movie clip				Menne et al., 2016		
**Product and/or software development**						
- Tutoring program/system					Graesser et al., 2006	
- Self-service checkout						Martin et al., 2013
**Reliability Of FACS**						
- Multiple						Sayette et al., 2001
**Sensory modalities and emotion**						
- Flavor and/or taste solutions		Bredie et al., 2014				Rosenstein and Oster, 1988; Bezerra Alves et al., 2013; Zacche Sa et al., 2015
- Multiple			Weiland et al., 2010	Greimel et al., 2006		
**AUTOMATIC**						
	**Implicit externally-reported measures**	**Explicit externally-reported measures**	**Vocalized self-reported measures**	**Non-vocalized self-reported measures**	**Combination**	**None**
**Affective states during learning**						
- Tutoring program/system		Grafsgaard et al., 2014				Grafsgaard et al., 2013
- E-book and/or audio book						Hung et al., 2017
**Behavior toward stimuli under social and/or situational contexts**						
- Computer game						Rossi, 2013
**Product and/or software development**						
- Food						Gurbuz and Toga, 2018
- Multiple						Brown et al., 2014
- Robot	Tussyadiah and Park, 2018			Bartlett et al., 2005		Gunes et al., 2019
- Toy						Espinosa-Aranda et al., 2018
**Sensory modalities and emotion**						
- Flavor and/or taste solutions						Chapman et al., 2017
**MANUAL** **+** **AUTOMATIC**						
	**Implicit externally-reported measures**	**Explicit externally-reported measures**	**Vocalized self-reported measures**	**Non-vocalized self-reported measures**	**Combination**	**None**
**Facial behavior and emotion expression**						
- Comedy, joke, and/or cartoon					Cohn et al., 2002	
**Product and/or software development**						
- Tutoring program/system		D'Mello and Graesser, 2010				
- Film and/or movie clip				Kodra et al., 2013		
- Multiple					Zhang et al., 2016	

### Facial Action Coding System (FACS)

In most studies (40 out of 55; 73%), participants affective responses were exclusively coded manually using FACS. Only 11 studies (20%) exclusively coded FACS automatically, of which 4 studies utilized the Computer Expression Recognition Toolbox (CERT), 6 studies used a novel software developed by the researchers, and 1 study utilized Affdex for Market Research by Affectiva. A total of 4 studies combined manually coding FACS with another measure that automatically coded FACS to determine participants' affective responses. For all 55 studies in this review, facial expressions were coded from pre-recorded and/or live video recordings.

For the studies that exclusively coded FACS manually, a majority (26 out of 40; 65%) utilized two individual coders to determine the participants' affective responses. However, some studies used one (5 studies), three (6 studies), four (1 study), and even six people (1 study) to manually code FACS. In the study by Catia et al. (2017), it was unclear how many individuals were utilized to code the human participants' facial responses. The purpose of these studies varied, but the majority investigated human development and/or behavior (10 studies; 25%). Though the stimuli of these studies were numerous, the majority of manually coded studies used comedies, jokes, and/or cartoons (7 studies; 18%) or film and/or movie clips (7 studies; 18%) to evoke an affective response from participants. Notably, the majority of these studies (22 out of 40; 55%) did not use another measure to validate FACS-determined affective response even though it has been suggested that there are many facets to evoking emotion and any single measure would fail to capture these facets in their totality (Lagast et al., 2017; Kaneko et al., 2018).

In most of the studies where FACS was exclusively coded automatically (6 out of 11; 55%), participants engaged with consumer product-based stimuli that were presented on a digital device (i.e., computer or computerized mobile device) including videos, online tutoring programs, computer games, and e-books. However, in three studies (27%), the consumer product-based stimuli themselves (e.g., robot or animatronic toy doll) contained the automatic FACS coding software. The purpose of some studies focused on product and/or software development (6 studies; 55%) whereas others investigated affective states during learning (3 studies), behavior toward stimuli under social and/or situations contexts (1 study), and relationship between sensory modalities and emotion (1 study). Similar to the manually coded studies, the majority of the automatically coded studies (8 out of 11; 73%) did not use another measure to validate FACS-determined affective response.

Of the studies that used both manual and automatic FACS coding, the majority (3 out of 4) focused on the development of products and/or software. Compared to studies that utilized manual or automatic coding alone to determine participant affective responses, all four of the studies that used both FACS applications validated FACS-determined affect with at least one and/or a combination of other methods. Though the methods varied, they encompassed all of the validation categories presented in this review (except “None”).

### Purpose of Study

An expansive variety of purposes were present in the selected studies (Table 2). For this review these purposes were categorized into a total of 9 typology-based categories: affective states during learning, behavior toward stimuli under social and/or situational contexts, facial behavior & emotion expression, genetics & emotion expression, human development and/or behavior, product and/or software development, reliability of FACS, and sensory modalities & emotion. In general, the most common purpose was to investigate development of products and/or software (11 studies; 20%). As over half of studies (28 studies; 51%) focused on human behavior in some respect, including the ones that investigated behavior under social and/or situational contexts (10 studies), facial behavior & emotion expression (eight studies), and human development and/or behavior (10 studies), it can be deduced that using FACS is considered a valuable tool for comprehending the impact of emotions on human appraisal as well as action tendencies. Nevertheless, some of the reviewed studies investigated the underpinnings of emotion and their relationship to human physiological capacities. In two studies, Haase et al. (2015) examined the effect of the short allele of the 5-HTTLPR polymorphism in the serotonin transporter gene on positive emotional expressions measured by objectively coded smiling and laughing behavior in response to cartoons as well as an amusing film. Cohn et al. (2002) assessed individual differences in rates of positive expression and in specific configurations of facial action units while participants watched short films intended to elicit emotion, which showed strong evidence that individuals may be accurately recognized on the basis of their facial behavior suggesting that facial expression may be a valuable biometric. Participants in the study by Weiland et al. (2010) were exposed to basic tastes (bitter, salty, sweet, sour, umami) as well as qualitatively different odors (banana, cinnamon, clove, coffee, fish, and garlic) while their taste- and odor- specific facial expressions were examined. Spontaneous facial expressions were also examined in response to distaste (Chapman et al., 2017) and to investigate whether they would provide additional information as to the explicit measure of liking (de Wijk et al., 2012) for basic tastes. As mentioned previously, FACS-measured facial responses were frequently utilized to develop various consumer products and/or software (11 studies; 20%). For a more detailed overview of the studies categorized by purpose and consumer product-based stimuli, see Table S3. The type of purpose varied widely across consumer product-based stimuli; the greatest number of stimuli by purpose were for flavor and/or taste solutions (*n* = 7, sensory modalities and emotion). Given the aforementioned variety of purposes for affective response measurement, it appears that FACS can be applied within numerous scientific fields.

### Consumer Product-Based Stimuli

A wide range of consumer product-based stimuli were used in the selected studies (Table 2). For this review, these products were categorized into a total of 18 product categories: amusement park ride; animal; cigarettes; comedies, jokes, and/or cartoons; computer games; e-book and/or audiobook; film and/or movie clip; flavor and/or taste solutions; food; gift/present; mobile application (app); multiple (i.e., more than one product-based stimuli); odor; physical game; robot; self-service checkout; toy; and tutoring program and/or system. A more detailed description of the stimuli used in each study can be found in the “Notes” column of Table S3. Although it could be argued that flavor or odor themselves are not consumer product-based stimuli, the reviewers deemed them appropriate categories since flavor and odor are inherent properties of purchasable consumer-product based stimuli (e.g., diffusible scents such those found in essential oils, candles, room or fabric fresheners, line-extension flavor varieties, etc.) that can elicit an affective response by evoking at least one of the five senses (i.e., taste or smell), which is in accord with the inclusion criteria of this review. In general, the most used product categories were films and/or movie clips (10 studies of which 8 featured only film and/or movie clips and two featured film and/or movie clips and another stimulus category) and comedies, jokes, and/or cartoons (nine studies of which 8 featured only comedies, jokes, and/or cartoons and 1 featured comedies, jokes, and/or cartoons and another stimulus category). In this review, most studies focused on utilizing consumer product stimuli that are emotionally competent (i.e., have the known and/or defined capacity to evoke a particular affective response) in nature, such as film clips and comedies. Similarly, flavor and/or taste solutions (seven studies) and odors (six studies) were also used as the single stimulus or one of multiple affect-evoking stimuli since certain tastes are emotionally competent, such as bitter, which elicits disgust motivated by withdrawal from products that may be harmful if consumed (Rozin et al., 1999).

Nevertheless, some of the reviewed studies investigated product categories with low, unknown, or undefined emotional competence. Some articles (three studies) used computer games to evoke adults' (Rossi, 2013; Balzarotti et al., 2014) as well as children's (Mui et al., 2017) affective responses in order to investigate their behavior under social and/or situational contexts. Tutoring programs/software were used in several studies to evoke an affective response in order to study affective states during learning (four studies) or to develop products and/or software (two studies). Grafsgaard et al. (2011), Grafsgaard et al. (2013), Grafsgaard et al. (2014) used either manual or automatic FACS coding to determine students' affective responses, while being tutored through an online program, because affective states often influence progress on learning tasks, resulting in positive or negative cycles of affect that impact learning outcomes (Woolf et al., 2009; Baker et al., 2010; D'Mello and Graesser, 2014). Participants of studies conducted by Sayette and Hufford (1995), Sayette and Parrott (1999), Sayette et al. (2001), Sayette et al. (2019), Griffin and Sayette (2008) and Sayers and Sayette (2013) underwent smoking cue exposure with cigarette and rolled-paper stimuli to better understand the relationship between affect and human behaviors including craving and urge. Children's facial expressions of affect were also manually coded in response to physical games to better understand human psychological development (Unzner and Schneider, 1990; Schneider and Josephs, 1991). Although food, used in studies by Forestell and Mennella (2012) as well as Gurbuz and Toga (2018), is recognized as emotional stimuli, researchers debate whether the nature of specific foods have the capacity to elicit intense emotional responses (Desmet and Schifferstein, 2008; Walsh et al., 2017a,b).

As such, studies with multiple stimuli seem to utilize products of known and/or high emotional competence alone or in combination with products of low, unknown, and/or undefined emotional competence in combination. For studies where multiple products were utilized, the number of stimuli varied widely: studies that analyzed pre-existing or pre-recorded video clips (of participants engaging with a consumer product-based stimulus) typically used between 2 and 5 stimuli whereas studies that presented products for participants to physically engage with in the present varied between 2 and 10 stimuli. Studies using multiple stimuli also featured some of the most underrepresented (≤ 2 studies) product categories in this review including amusement park rides, animals, e-books and/or audio books, gift/present, and mobile application (app). Though the study by Brown et al. (2014) was marked as having multiple stimuli, it was marked as such because it was unclear if the stimulus used is an actual e-book or a mobile application (app) featuring an e-book. Based on previous suggestions for emotional research by King and Meiselman (2010) the number of products tested for emotional measurement is important because it can increase the number of statistically significant differences in measured emotions.

### Validation of Emotion

#### Method Type

Though method of affect validation was an outcome of interest in the review, more than half of the included articles (29 out of 55 studies; 53%) did not employ additional methodology to validate FACS-determined affect. Non-vocalized self-reported methods were most commonly applied and appeared in a total of 14 articles (25%) as either the sole validation method (11 studies) or in combination with other methods (four studies). In Table 2, all studies are categorized by type of method used to measure emotion. Following the typology (see Table S4), the methods are further classified by type of measure/instrument used to validate FACS-determined affect. A more detailed description of the validation measures used in each study can be used in each study can be found in the “Notes” column of Table S3. For the explicit externally-reported method, five type of measures were found: manual FACS coding, gross body movements, vocal quality cues, and subjective human judgment of emotion. Also, seven measure categories for the implicit externally-reported method were listed: skin conductance/electro dermal activity, heart rate (HR), electrocardiogram (ECG), facial electromyography (EMG), EEG, respiratory sinus arrhythmia, respiratory rate (RR), and blood pressure (BP). For the non-vocalized self-reported method, five types of measures were identified: dial reporting, Likert-type scales for emotion and emotion intensity rating, positive and negative affect schedule (PANAS) questionnaires, mood state questionnaires, and Likert scales for pleasantness. Also, two measure categories for the vocalized self-reported method were recognized: vocalized/vocal report of emotion and vocalized/vocal report of pleasantness. For the studies that combined validation methods, five categories were identified: explicit externally-reported & vocalized self-reported, implicit externally-reported & vocalized self-reported, explicit non-vocalized self-reported, implicit externally-reported & non-vocalized self-reported, and explicit & implicit externally-reported.

#### Explicit Externally-Reported Measures (Nine Incidences Reported)

Of the 55 studies included in this review, only eight studies (15%) reported use of explicit externally-reported methodology: four articles where the method was the only validation method and 4 articles where the method was used in combination with other methods. Explicit externally-reported measures of affect validation were most frequently applied through subjective judgement of emotion (four studies) and tracking of gross body movements (three studies), both of which were performed by a human individual that was neither the subject nor the study's principle investigators. For articles where the subjective human judgement of emotion was employed (four studies), FACS was coded manually (three studies) or automatically (one study) and it was always combined with another method and/or measure. Of the studies using gross body movements to validate affective responses, FACS was automatically coded (one study), manually coded (one study), as well as coded both manually and automatically (1 study). Vocal quality cues, coded by individuals trained to use the maximally discriminative facial movements coding system (MAX), were another explicit externally-reported measure used in combination for affect validation (1 study). Bartlett et al. (2005) used subjective judgement by observers using the turn dial technique to rate amount of happiness shown by the subject. Notably, one study by Bredie et al. (2014) manually coded facial expressions using FACS and utilized manually coded facial expressions from a previous study by the same group to validate the affective responses they measured. In most studies, the timing of the explicit externally-reported validation measurement took place concurrently (five studies) or after (three studies) facial expressions had been coded using FACS.

#### Implicit Externally-Reported Measures (15 Incidences Reported)

Of the 55 studies reviewed, only seven studies (13%) used implicit-externally reported methods: 1 article where the method was the only validation method and six articles where the method was used in combination with other methods. The registration of HR (five studies) was most popular and occurred in studies where implicit-externally reported measures were the exclusive validation method (1 study) as well as when this method was combined with other methods (four studies); in this review, heart rate was usually accompanied by another implicit-externally reported measure (five studies) such as skin conductance, blood pressure, etc. For studies that used implicit-externally reported methods, facial expressions were mostly coded manually using FACS (4 studies) but were also coded automatically (1 study) or by using both manual and automatic coding (two studies). Only two studies exclusively used a single implicit externally-reported measure (Facial EMG = 1 study, EEG = 1 study) whereas five studies employed at least 2 or more implicit externally-reported measures including HR (5 studies), ECG (1 study), skin conductance/electrodermal activity (three studies), RR (two studies), respiratory sinus arrhythmia (1 study), and BP (1 study) to validate FACS-determined affect. In these studies, facial expressions occurred concurrently with the measurement of implicit-externally reported methods.

#### Vocalized Self-Reported (4 Incidences Reported)

Of the 55 studies reviewed, only five studies used vocalized self-reported methods, either vocalized report of emotion or vocalized report of pleasantness. Verbal/vocal report of emotion was most common (four studies) and was used exclusively as the affect validation measure (1 study) or in combination with another method (three studies) and was utilized only when facial expressions were manually coded with FACS. Vocalized report of pleasantness (1 study) was only reported once in this review and was the only measure used to validate manually coded, FACS-determined affect in that study. In studies that used vocalized self-reported methods, facial expressions occurred concurrently (three studies) or before (two studies) the vocalized self-report validation method.

#### Non-vocalized Self-Reported (16 Incidences Reported)

Of the 55 studies reviewed, only 14 studies (25%) used non-vocalized self-reported methods: 10 articles where the method was the only validation method and four articles where the method was used in combination with other methods. The rating of affect and its intensity on rating scales (eight studies) was most popular in the non-vocalized self-reported validation method, which occurred most often in the form of 9-point (3 studies), 7-point (3 studies), and 5-point (2 studies) Likert-like scales; however, in Jakobs et al. (1999), subjects rated intensity of nine emotional feelings (interest, happiness, boredom, surprise, contentment, irritation, excitement, amusement, and disgust) on 100-millimeter rating scales numbered at each centimeter, in which 0 = not at all and 100 = extremely. Mood (1 study) or affective state (two studies) questionnaires were also utilized by subjects to indicate the “state” they were in while engaging with a stimulus. Two studies used the PANAS questionnaire which consists of a number of words that describe different feelings and emotions where subjects indicate to what extent they have felt in the present moment or over a particular period of time (e.g., the past week). In another (1 study) by Soussignan and Schaal (1996), subjects' hedonic ratings were assessed using five differently colored cards, each bearing a label describing a hedonic tone (from 1 = very unpleasant to 5 = very pleasant) and subjects were asked to point to the card that best fit with their assessment of how pleasant the stimuli were. However, it should be noted that these questionnaires, scales, and rating measures are discontinuous and only capture affect as it holistically relates to an experience. Additionally, a few articles (two studies) utilized dial reporting—a more continuous measure where subjects are asked to turn a hardware dial to quantify valence throughout their engagement with a stimulus; e.g., on a 0–100 dial range, subjects are typically told that 0 is disinterest, 50 is neutral, and 100 is interest in the stimulus. For the studies that utilized non-vocalized self-reported validation methods, the majority (12 studies) coded facial expressions manually using FACS whereas the minority (two studies) used both manual and automatic coding.

#### Studies Combining Validation Methods

Of the eight studies (15%) that combined validation methods and used multiple validation measures, the combinations fell into the following categories: explicit externally-reported & vocalized self-reported (two studies); explicit externally-reported & non-vocalized self-reported (1 study); implicit externally-reported & non-vocalized self-reported (1 study); explicit externally-reported & implicit externally-reported (1 study); implicit externally-reported, vocalized self-reported, & and non-vocalized self-reported (1 study); and explicit externally-reported, implicit externally-reported, and non-vocalized self-reported (1 study). The study by Cohn et al. (2002) combined the implicit externally-reported method (measure= Facial EMG) with a self-report of emotion; however it was unclear if the self-report was vocalized or non-vocalized. Likewise, it was also unclear if subjects' facial expressions occurred concurrently or before the self-report of emotion. For the studies that utilized a combination of validation methods, the majority (6 studies) coded facial expressions manually using FACS whereas the minority used both manual and automatic coding (two studies).

### Risk of Bias

Of the 55 studies included in this review, 11 studies (20%) were determined to have high risk of bias in one or more of the following categories: selection bias (allocation concealment, sequence generation), performance bias (blinding of participants and personnel to all outcomes), detection bias (blinding of assessors to all outcomes), attrition bias (incomplete outcome data), reporting bias (selective outcome reporting), and other sources of bias. One study was identified as being at risk of selection bias because the videos the experimenters used were shown in the same order and it was unclear if the conditions (solitary or social interactions) were randomized (Frank et al., 1997). The risk of performance bias was judged to be minimal since blinding of participants and personnel was determined to be low (38 studies) or unclear (17 studies). Detection bias was a concern for a few studies (five studies) as the accessors were not blinded to all study outcomes. In the study by Brown et al. (2014), it appears that the evaluation of experimental treatments/stimuli were performed by the prototype developers (i.e., the authors). Similar circumstances were presented in the study by D'Mello and Graesser (2010) where the two FACS coders “had considerable experience interacting with AutoTutor. Hence, their emotion judgments were based on contextual dialogue information as well as the FACS system.” Likewise, three studies identified that the FACS coder(s) was one of the principle investigators of that study and, therefore, was aware of the study's aims (Lynch, 2010; Lynch and Trivers, 2012; Martin et al., 2013). Attrition bias was also a concern for a few studies (four studies) as there was incomplete outcome data. In the study by Brown et al. (2014), the authors did not clearly state their outcomes of interest and, though they discussed some of the main areas of work they performed, the criteria they evaluated the prototype for was not well-defined or discussed. The study by Cohn et al. (2002) listed comparisons of facial behavior with self-reported emotion, but no methodology is outlined for how self-reported emotion was measured nor for any statistical analysis being performed; although the experimenters later reported correlations between self-reported emotion and zygomatic major activity, no comparisons were made for self-reported emotions and AUs. Similarly, Frank et al. (1997) rating dial and subject self-report data was not included in the study's results or discussion. Additionally, Tussyadiah and Park (2018) reported using the Emotion Facial Action Coding System (EMFACS) to score emotion expressions (joy, anger, surprise, fear, contempt, sadness, and disgust) but no scores were presented for each emotion in the results; only percent occurrence of the emotional expression was discussed and graphically depicted. Reporting bias was a concern for a few studies (six studies) as there was selective outcome reporting. For four studies, the outcomes listed in the methods section of the article could not be compared with those whose results were reported (Frank et al., 1997; Cohn et al., 2002; Bezerra Alves et al., 2013; Brown et al., 2014). Also, in the study by Catia et al. (2017), the authors compared the human videos coded with FACS to the established literature instead of a within-study control for reliability, which could lead to selection of literature that supports their findings instead of collectively reporting on the entire body of evidence that exists. In the study by Gurbuz and Toga (2018), the researchers cited that the automatic FACS coding software they developed scored basic emotions (anger, contempt, disgust, fear, happiness, neutral, sadness, and surprise) from 0 to 1 but only reported that the expression happiness was a significant predictor of gender; no emotion scores were reported. Other sources of bias were found in three separate studies. Brown et al. (2014) made several conclusions about their e-books abilities that were not supported by the data presented, which could lead to conclusions that are not realistic or substantiated by the data. In the videos selected by Catia et al. (2017), there is no justification or verification of the subjects' emotional experience; therefore, the authors may be speculating that a particular emotion is being expressed when it really is not. In which case, their authors' basis for the specific emotional competency of each stimuli may be false and subsequent interpretations of the data may be invalid. Additionally, Frank et al. (1997) utilized data from a previously conducted study, which may contribute to other potential sources of bias, and also mentioned the removal of outlier data—a practice generally considered to be unethical.

## Discussion

This is the first study that systematically reviews the purpose and validation of (manual, automatic, and a combination of both manual and automatic) FACS implementation for the measurement of human emotional behavior to different types of consumer product-based stimuli.

It provides a comprehensive overview on 55 peer-reviewed articles published between 1978 and June 2019 and builds on the increased interest in the relationship between emotions and consumer-based products, which goes beyond sensory liking, by indicating trends of capturing and validating affective responses with FACS. The main observations were: (1) The vast majority of studies neglected to employ additional methodology to validate FACS-determined affect (29 out of 55 studies; 53%); (2) Of the validation measures that were used, most tended to be discontinuous in nature and only captured affect as it holistically related to an experience; (3) As described in the articles' methodologies, researchers typically utilized consumer product-based stimuli that had known and/or defined capacity to evoke a particular affective response, and a lack of attention was paid to consumer products with low levels of emotional competence or with unknown affective impact. Additionally, this review illuminated some inconsistencies in how FACS is carried out as well as how affective (i.e., emotional) response is inferred from AU activation—these inconsistencies will be outlined and recommendations will be suggested to enhance the interpretation of emotion as it pertains to consumer product-based stimuli.

### Neglecting to Validate FACS-Determined Affect

Emotions are considered to be important drivers of consumer product-related perceptions such as liking and preference. Valid, reliable, and sensitive instruments that assess consumer product-elicited emotions are therefore valuable for fundamental and applied research, developing new products, and advocating for a healthy lifestyle and/or behavior. As mentioned in the results, the fact that at least half of the studies in this review failed to employ an additional validation measure makes it evident that researchers assume that FACS is an accurate and reliable approach for assessing product-evoked emotions. FACS has been used to verify the physiological presence of emotion in a number of studies, with high (over 75%) agreement (Bartlett et al., 2005), and good to excellent reliability for the occurrence, intensity, and timing of individual action units and for corresponding measures of more global emotion-specified combinations (Sayette et al., 2001). Although FACS is an ideal system for the behavioral analysis of facial action patterns and humans can be trained to code reliably the morphology of facial expressions (which muscles are active), it is very difficult for them to code the dynamics of the expression (the activation and movement patterns of the muscles as a function of time) (Bartlett et al., 2005). Additionally, human emotion is a multifaceted construct linked to physiological, behavioral, and cognitive processes, and we may not assume to find a single measure that covers the full range, although there is a general agreement that all measures are relevant (Ruch, 1997a,b). Thus, it can be concluded that emotions (i.e., an affective response) evoked by consumer product-based stimuli can only be fully-understood by integrating data from multiple modalities (e.g., facial expressions, physiological responses, self-report).

### Tendency Toward Discontinuous Validation Measures

Of the 19 validation measures reported, there were 14 instances of non-vocalized self-reported, 15 instances of implicit externally-reported, 10 instances of explicit externally-reported, and 4 instances of vocalized self-reported measures. Regarding these incidences, the non-vocalized self-reported measure was by far used in the most articles (14 studies) with 10 of those studies using the method as the only measure of validation. As previously mentioned, the majority (more than 86%) of measures used in the non-vocalized self-reported method are discontinuous measures of affect such as questionnaires, scales, and rating procedures; the only continuous measure reported was the dial reporting method. Discontinuous measures existed in other methods as well-including subjective human judgement of emotion (explicit-externally reported), vocalized/vocal report of emotion (vocalized self-report), and vocalized/vocal report of pleasantness (vocalized self-report). There are two important comments to be made here. First, report of product engagement can vary over time in emotional response due to perceptual variability, changing expectations, and preceding contexts. Most studies, especially when self-reported methods were used, measured emotion after engagement with a consumer product-based stimulus. As such the method does not continuously measure emotional response alongside facial expressions, which can account for inconsistencies between the two reported affective states (Kodra et al., 2013). Externally-reported methods (implicit and explicit) can provide a measure of affect that is not encumbered by issues associated with self-reported methodology. Explicit externally-reported methods in this review were exclusively applied/measured by another human being including gross body movements, vocal quality cues, and subjective human judgement of emotion. While the latter is a discontinuous measure as it was performed after the initial product-engagement session, the former measures offer a more continuous report of affect as they occurred concurrently with facial expression of emotions. However, Graesser et al. (2006) noted that the humans applying these methods have varying levels of accuracy in their judgements, which were dependent upon the degree of training they had received and were subject to bias from human cognitive processes. Implicit externally-reported methods apply continuous monitoring of emotion measurement using instrumentation, which avoid the limitations of self-report and explicit externally-reported methods. In this review, the use of an implicit externally-reported measure (8 measures; 15 reported incidences) was mostly the result of interdisciplinary research as techniques from psychology and medical science were applied. FACS was developed by psychologists Ekman and Friesen (1976, 1978) because their research supported that the face may also influence a person's emotional experience by providing signals to others about how the person feels and; thus, FACS is frequently used in the field of psychology. Similarly, the measurements of autonomic nervous system (ANS) responses (such as heart rate) and neurophysiological responses (such as brain activity) originate from psychophysiology and have only recently been applied in consumer and sensory research (Bradley, 2000; Bradley et al., 2001; Codispoti et al., 2006, 2009). According to Zhang et al. (2016), research has also shown a correlation between the physiological state to the emotional state of individuals. Based on the studies in this review that incorporated implicit measures to validate FACS-determined affect, results indicate that the combined use of implicit measures can yield super additive effects for interpretations of some affective states but redundant and inhibitory effects for others.

### Researchers' Proclivity for Emotionally-Competent Stimuli

Although authors report FACS and affective data evoked by a variety of consumer product-based stimuli, these are frequently of a category and/or include a stimulus that has a known and/or defined capacity to evoke a particular affective response: films and/or movie clips, comedies, jokes, and/or cartoons, and flavor/taste solutions dominated as the stimuli of choice in the reviewed studies. It seems that this is a result of the nature of the reviewed studies as the majority (28 studies; 51%) investigated human behavior in some context including: affect in human development & behavior (10 studies;18%), behavior toward stimuli under social and/or situational contexts (10 studies; 15%), as well as facial behavior & emotion expression (8 studies; 15%). In these cases, the researchers desired to induce a particular affective state or elicit a specific emotion from subjects so they could understand its impact on their behavior. The existing body of literature suggests that emotional and motor processes are strongly interrelated, but attempts to assess whether emotionally-competent stimuli (i.e., stimuli with known and/or defined capacity to evoke a particular affective response) modulate action readiness (i.e., the ease with which an action may be initiated given the pre-action state an individual) has yielded contradictory results due to differences in experimental design (Avenanti et al., 2005; Coombes et al., 2005; Hajcak et al., 2007; Schutter et al., 2008; van Loon et al., 2010). To overcome these contradictions, Mirabella (2018) devised a new version of an emotional go/no-go task to directly compare equivalent decision-making processes underlying actions cued by emotional stimuli of differing valence. In their work, it was demonstrated that only when the emotional content of the stimuli was relevant for the task did it impact the generation of actions (Mirabella, 2018). As such, if task-relevance is a crucial factor, then consumer response to an emotionally-competent stimulus would not be directly related to the stimulus itself; rather it would be contingent on the cognitive state of the consumer.

Several studies referenced or characterized the relationships between flavors, odors, and the specific affective responses they evoke (Rosenstein and Oster, 1988; Greimel et al., 2006; Weiland et al., 2010; Bezerra Alves et al., 2013; Bredie et al., 2014; Zacche Sa et al., 2015; Chapman et al., 2017). Notably, only two in this review sought to use FACS to assess consumer engagement for the purpose of understanding preference for (1 study) or acceptability of (1 study) a consumer-based product (Lynch, 2010; Forestell and Mennella, 2012). The scientific need to better conceptualize consumers' experience with products of low or unknown/undefined capacity to evoke an affective response (such as product prototypes, line extensions, etc.) has led to an increased interest in integrating emotions into consumer and sensory research. Several articles (11 studies) in this review utilized FACS for the purpose of product and/or software development, however, the majority (six studies) were focused on developing and/or validating software or consumer-based products that contained software for automatic recognition of facial expressions of affect; only three studies were truly using FACS to assess consumer affective response to product-based stimuli that were being developed including self-service checkouts and robots (Martin et al., 2013; Tussyadiah and Park, 2018; Gunes et al., 2019). In recent years, there has been a particular focus on the relationship between food and emotions for the sake of understanding food-evoked affect on acceptability, intention to purchase, food choice, attitudes, or behavior, which has led to the introduction of many methods and measures to capture consumers' emotions elicited by food (Lagast et al., 2017; Kaneko et al., 2018). Much like traditional sensory methods already in use by the industry for acceptance or preference, explicit methods of emotion measurement rely on participant cognition of and ability to recognize affective [emotional] response. However, a lack of emotion term understanding as well as pre-existing attitudes and stereotypes for products causes high panelist-to-panelist variation and within-panelist inconsistency (Leitch et al., 2015; Walsh et al., 2017b). Likewise, the complexity and lack of specificity of implicit methods makes emotion interpretation incredibly challenging. Although research suggests that implicit measures are likely sensitive enough for consumer products with intensely polarized (very high or very low) liking, some studies have even concluded that implicit measures such as ANS responses and facial expressions cannot reliably differentiate sensory effects and changes in emotional valence (de Wijk et al., 2012; Danner et al., 2014a,b; Walsh et al., 2017a). It has also been suggested that context can be an important and influential source of information when inferring emotional meaning in a facial configurations (Carroll and Russell, 1996). As such, it can be deduced that the emotions elicited by products are often undefined and researchers should increasingly implement a variety of affective [emotion] measurement methods and integrate it with contextual data to provide a more comprehensive understanding of consumers' preferences and drivers of product-related choices.

### Procedure for Utilization of FACS

FACS is the most comprehensive, psychometrically rigorous, and widely used system to describe facial activity in terms of visually observable facial muscle actions (i.e., AUs). Because of its descriptive power, FACS is regarded by many as the standard measure for facial behavior and is used widely in diverse fields such as neuroscience, computer vision, computer graphics and animation, facial encoding for digital signal processing, behavioral science, and psychology (Cohn et al., 2007). This review shows that there are many inconsistencies with how researchers utilized FACS, both manually and automatically, to measure emotion. The main areas of inconsistency pertained to participant awareness of their facial expressions being video recorded, training of the coder and/or coding system, and validation of coder or automatic coding system's ability.

Whether manually or automatically applying FACS, video-recorded facial behavior must be captured and exhaustively analyzed frame-by-frame. For studies in this review, 55% (30 studies) reported participants being aware of the camera and/or that they were being video recorded, 25% (14 studies) were unaware because of camera concealment (8 studies) or because the participants were infants (six studies), and 20% (11 studies) did not identify or make clear whether or not participants were cognizant of being video recorded. Most often, participants were made aware of the video recording as part of the informed consent process and, depending on the transparency level, this process may have informed participants that the recordings would be used to analyze their facial expressions. Consequently, any resulting data and interpretations could be negatively influenced by subject reactivity bias; participants are cognizant that their actions and reactions are being closely observed, which may cause them to exaggerate or moderated facial expressions (Weiland et al., 2010; Jewitt, 2012). To reduce the potential for subject reactivity bias, researchers should employ a consent process that informs panelists that the experiment will be continuously video recorded but should not disclose that their facial expressions would be analyzed.

As previously mentioned, this review illuminated inconsistencies related to coder (manual and automatic) training and validation. For studies that employed manual coding (44 total; 40 used manual only, 4 used manual in combination with automatic coding), 67% (29 studies) identified that the coders were certified-trained in FACS while 34% (15 studies) failed to identify if one or all coders were FACS certified-trained. Barrett et al. (2019) maintain that people's capacity to reliably perceive emotions in the “common view” expressive configurations depends on how participants are asked to report or register their inferences. Likewise, there appears to be important cultural variation in whether emotions are perceived as situated actions or as mental states that cause actions (Barrett et al., 2019). Although FACS is widely considered to be an ideal system for the behavioral analysis of facial action patterns, the process of manually applying FACS to videotaped behavior has been identified as a major obstacle (Bartlett et al., 2005). To become FACS certified-trained, one must become intimately familiar with the content of the 597-page self-instructional text (also available on compact disk, CD), as well as the 197-page Investigator's Guide; at the time this review was published, FACS preparation materials are priced at $350 (Paul Ekman Group LLC, 2019). The time required to learn FACS is variable, depending on a number of factors including number of hours per week that can be devoted to training, the availability of expert trainers, and individual differences among trainees. Cohn et al. (2007) approximate that it would take 3 months to become proficient so as to demonstrate mastery on the FACS Final Test, which is a 34-question video-based exam priced at $50 (Paul Ekman Group LLC, 2019). It then takes, on average, over 2 h to comprehensively code 1 min of video. Furthermore, although humans can be trained to reliably code the morphology of facial expressions it is very difficult for them to code the expression dynamics (i.e., movement patterns of the muscles as a function of time) as well as the occurrence of micro expressions, which occur at very high frequencies (1/15th−1/25th of a s) (Bartlett et al., 2005)—even FACS certified-trained individuals need to undergo a separate specific training to reliably code micro expressions. Without rigors of becoming FACS certified trained, researchers attempting to use FACS may do so with varying levels of reliability. Likewise, researchers have a higher likelihood of success when one or more of their coders has demonstrated validated reliability via their FACS Final Test (following submission of the test scores, a trainee receives a reliability measure of their score compared with those of experts in FACS scoring) and/or by calculating interrater reliability (e.g., Cohen's Kappa).

For studies that employed automatic coding (15 total; 11 use automatic only, 4 used automatic in combination with manual coding), 73% (11 studies) identified that the coding system was trained and validated while only 27% (four studies) failed to identify if the system had been trained and/or validated. Similar to manual coders, automatic coding systems require training to ensure reliable coding of AUs. This training is performed by having the system evaluate still images and/or videos from a database where the facial AUs being activated (and emotions being expressed) are fully coded and comparing the system's determinations with the database. Based on this review, the automatic systems used for analysis were trained on a variety of databases including those that contained still images of posed facial expressions (e.g., Pictures of Facial Affect, POFA; Psychological Image Collection at Stirling University, PICS; MMI-Facial Expression Database; Cohn-Kanade DFAT-504) as well as those that contained videos depicting spontaneous facial behavior (e.g., Cohn-Kanade AU-Coded Expression Database, CK+; Rutgers and University of California San Diego FACS database, RU-FACS; Binghamton-Pittsburgh 3D Dynamic Spontaneous Facial Expression Database, BP4D). Although most automatic systems were trained on a spontaneous expression database, several did not identify how their system was trained (D'Mello and Graesser, 2010; Hung et al., 2017; Gurbuz and Toga, 2018; Tussyadiah and Park, 2018) or were solely trained on posed still image databases (Brown et al., 2014; Gunes et al., 2019), which may put their interpretations of participant emotion responses to consumer product-based stimuli at risk. Spontaneous facial expressions differ substantially from posed expressions; subjects often contract different facial muscles when asked to pose an emotion such as fear (subjects perform AUs 1+2), vs. when they are actually experiencing fear (spontaneous fear reliably elicits AUs 1+2+4) (Ekman, 2009). Additionally, spontaneous expressions have a dynamically fast and smooth onset in which muscle contractions in different parts of the face peak at the same time. In contrast, posed expressions tends to be slow and jerky, and the muscle contractions typically do not peak simultaneously (Bartlett et al., 2005). Thus, researchers attempting to use the automatic FACS coding may do so with varying levels of reliability unless their system has been trained and validated against at least one, if not more, spontaneous expression databases.

### Relationship Between Action Units and Emotion Expression

Although FACS itself is descriptive and includes no emotion-specified descriptors, it is commonly used to interpret non-vocalized communicative signals, such as facial expressions related to emotion or other human states (Valstar and Pantic, 2006). Using FACS, human observers can uniquely break down a facial expression into one or more of AUs that comprise the expression in question including: nine action units in the upper face and 18 in the lower face. In addition, there are 14 head positions and movements, nine eye positions and movements, five miscellaneous action units, nine action descriptors (i.e., movements for which the anatomical basis is unspecified), nine gross behaviors, and five visibility codes (Ekman et al., 2002). If one wishes to make emotion-based inferences from single and/or combinations of AUs, a variety of related resources exist such as EMFACS and the FACS Investigators' Guide (Ekman et al., 2002), the FACS interpretive database (Ekman et al., 1998), and a large body of empirical research (Ekman and Rosenberg, 2005). With respect to the studies in this review, there were inconsistencies with how affective (i.e., emotional) response is inferred from AU activation (Table S6). Specifically, there were instances where AUs as well as emotion-based inferences were not used in agreement with FACS.

Of the 55 studies in this review, the majority (49 studies; 85%) reported coding AUs as they are defined in the FACS system (e.g., AU 12 = lip corner puller, *Zygomaticus major*). However, there were 2 studies (Rosenstein and Oster, 1988; Brown et al., 2014) where it was unclear if the AUs were coded as defined in the FACS system and 4 studies (Soussignan et al., 1999; Bezerra Alves et al., 2013; Kodra et al., 2013; Zacche Sa et al., 2015) that reported coding/defining the AUs in a manner that diverged from FACS. AU's were not identified in 4 studies in which the automatic emotion coding software was being developed/piloted to measure emotions in response to consumer products (Brown et al., 2014; Espinosa-Aranda et al., 2018; Gurbuz and Toga, 2018; Tussyadiah and Park, 2018). As suggested in the FACS Investigators' Guide (Ekman et al., 2002), it is possible to map AUs onto the basic emotion categories using a finite number of rules (Table 3). When researchers deviate from these rules and define AUs and emotions by their own criteria (e.g., AU12 = smile, lateral lip corner pull without AU04 or AU09), such as in the study by Kodra et al. (2013), their emotional interpretation has a higher chance of being faulty unless substantiated by a large body of empirical research. There are iterations of FACS, such as the Baby FACS, which are supported by existing literature. A few studies in this review reported using Baby FACS to code the facial expressions of affect in infants (Soussignan et al., 1999; Bezerra Alves et al., 2013; Zacche Sa et al., 2015). Rosenstein and Oster (1988) also reported using Baby FACS but the AUs they reported were more consistent with the adult FACS. Baby FACS is an anatomically based coding system adapted from Ekman et al. (2002) adult FACS that consists of the following action units: A1 = no distinct mouth action or sucking on the face; A2 = A1 with a negative expression on the mid-face; A3 = A1 with a negative expression on the mid-face and brows; B1 = pursing mouth; B2 is B1 with a negative expression on the mid-face; B3 = B1 with a negative expression on the mid-face and brows; C1 = mouth-gaping action; C2 = C1 with a negative expression on the mid-face; C3 = C1 with a negative expression on the mid-face and brows. Unless researchers are seeking to contextualize facial expressions of novel or undefined emotions, attitudes, or moods, such as when Grafsgaard and others were investigating facial expression of confusion, they should utilize the AU-specific rules outlined for the basic emotions as outlined in in the FACS Investigators' Guide.

**Table 3:** Rules for mapping Action Units to emotions, according to the FACS investigators guide. A/B means “either A or B”.

**Emotion**	**AUs**
Anger	4+5+7+10+22+23+25/26
	4+5+7+10+23+25/26
	4+5+7+17+23/24
	4+5+7+23/24
	4+5/7
	17+24
Disgust	9/10+17
	9/10+16+25/26
	9/10
Fear	1+2+4
	1+2+4+5+20+25/26/27
	1+2+4+5+25/26/27
	1+2+4+5
	1+2+5+25/26/27
	5+20+25/26/27
	5+20
	20
Happy	12
	6+12
Sadness	1+4
	1+4+11/15
	1+4+15+17
	6+15
	11+17
	1
Surprise	1+2+5+26/27
	1+2+5
	1+2+26/27
	5+26/27

Additionally, it should be mentioned that recent research refutes the common view that facial configurations are “fingerprints” or diagnostic displays that reliably and specifically signal particular emotional states regardless of context, person, and culture (Barrett et al., 2019). Instead, when facial movements do express emotional states, they are considerably more variable—rich in the variety with which people spontaneously move their faces to express emotions in everyday life. As such, it has been suggested that technology that applies facial expression nomenclatures & mapping constructs [such as the FACS and its AUs] fail to reliably interpret facial expressions of emotion.

### Limitations

Although this systematic review aimed to give an overview of how FACS has been used to investigate human emotional behavior to consumer product-based stimuli, the inclusion and exclusion criteria narrowed the search down to studies that reported using FACS and/or the AUs of FACS to measure and/or characterize emotion. As such, studies that utilized measures that were derived from and/or engaged FACS in their analysis of facial expressions, such as automatic facial expression analysis (AFEA) software but neglected to identify whether the AUs measured and/or characterized emotion were excluded. In the initial search, several studies used AFEA instruments that have the capacity to measure and output data on AU activation and intensity (e.g., FaceReader™) but they only reported emotion based on the software's algorithm-determined output. Because such studies were excluded from this review, there could be a variety of consumer product-based applications of FACS that went unaccounted.

## Conclusions

This review aimed to present how the facial action coding system has been used to investigate human emotional behavior to consumer product-based stimuli. While this field of research is rapidly growing, this systematic review offers a comprehensive overview of the purposes, applications, and methods of validating FACS-determined affective responses for different types of consumer product-based stimuli. Given the aforementioned variety of purposes for affective response measurement, it appears that FACS can be applied within numerous scientific fields. This review may prompt researchers to consider measuring the total consumer experience by employing a variety of methodology, in addition to FACS, to better conceptualize consumers' experience with products of low, unknown, and/or undefined capacity to evoke and affective response such as product prototypes, line extensions, etc. It must be noted that there are many more compounding factors that work to influence consumer choice and buying behavior in the market such as culture, geographical location, income, and individual experiences. However, utilizing a combination of measures, such as those that capture continuous as well as discrete emotional responses in both implicit as well as explicit contexts, is progressive step to better predict an individual's actual decision-making and affective response. Future research could review the results generated by the different FACS applications and validation measures in order to compare and evaluate them by consumer product type.

## Data Availability Statement

All datasets generated for this study are included in the article/Supplementary Material.

## Author Contributions

EC put forward the review idea, developed the inclusion & exclusion criteria, organized the article structure, and wrote the draft manuscript. EC and J'NK selected the databases to be searched, developed the search string, searched the relevant literature, read the literature, and identified the articles included in this review. SD assisted in conceptualizing the original question, reviewed and edited the drafted manuscript and provided critical guidance for integrating the introduction, results, and discussion topics. J'NK, MB, JL, DG, and SO'K contributed to identification of discussion topics for improving the interpretation of the observations from the systematic review, reviewed, and edited the manuscript.

## Conflict of Interest

The authors declare that the research was conducted in the absence of any commercial or financial relationships that could be construed as a potential conflict of interest.

## Supplementary Material

The Supplementary Materials of this manuscript include: Tables S1-S6. Table S1 contains the inclusion and exclusion criteria used for article selection. Table S2 outlines the application of the PICO (S, T) framework for this review. Table S3 depicts the data extraction sheet layout and contains all the extracted data for this systematic review. Table S4 outlines the typology for the purpose, stimuli, and validation method categories used in this review. Table S5 contains the definitions and interpretations of key words and frequently used terms in this systematic review. Table S6 outlines the AUs investigated and/or reported on and their identified emotional interpretation by study.
