# Supplementary material for: The Facial Action Coding System for Characterization of Human Affective Response to Consumer Product-Based Stimuli: A Systematic Review
Source: Front Psychol. 2020 May 26;11:920. doi: 10.3389/fpsyg.2020.00920 (PMC7264164; doi:10.3389/fpsyg.2020.00920)
Supplement: Supplementary file 1 [file Table_1.DOCX]

**Table S.1** Inclusion and exclusion criteria used for article selection

| *Inclusion*   - Full text articles written in English - Studies on humans - Studies where affective (i.e. emotional) response is elicited by consumer product-based stimuli, which evoke at least one of the five senses (sight, touch, smell, hearing, taste); research employs a sensory modality to induce an affective response - Studies that investigate affect (i.e. emotion) using the facial action coding system and/or the facial action units of the facial action coding system |
| --- |
| *Exclusion*   - Articles written in a language other than English - Studies conducted on animals - Studies that measure and or characterize affective response to media-based messaging, webpages, or forums used to market and/or sell consumer products - Studies that do not use the facial action coding system and/or the facial action units of the facial action coding system to measure and/or characterize emotion - Studies that included participants with eating disorders (i.e. anorexia nervosa, bulimia nervosa), neurologic, psychiatric, or physiological disorders |

**Table S.2** Application of the PICO (S, T) framework for this review

| **PICOS elements** | **Relevant search terms** | **Justification** |
| --- | --- | --- |
| **Population** | Consum*, Panel*, Participant, Customer, Client, Purchaser, User, Buyer, Shopper, Parton, Vendee | Limit to human population using database search limits option |
| **Intervention** | Facial Action, Automatic Facial Expression Analysis | Intervention of interest: characterization of emotion using the Facial Action Coding System (FACS) or Facial Action Units (AUs) of the FACS |
| **Comparator** | All study designs and comparisons | All study designs and comparisons are included |
| **Outcome** | All outcomes of interest identified. | Targeted outcomes: purpose, methodology (FACS application), consumer product-based stimuli, and validation of FACS-assessed consumer affect (i.e. emotional response) |
| **Time** | 1978-Present | Limit studies to those published from 1978-Present |

**Table S.3** Data extraction sheet layout containing all extracted data for this systematic review

| **Study**  **(author, year)** | **Title** | **FACS** | **Purpose of Study** | **Stimuli** | **Validation of Affect** | **Notes** |
| --- | --- | --- | --- | --- | --- | --- |
| Balzarotti et al. (2014) | “I know that you know how I feel”: Behavioral and physiological signals demonstrate emotional attunement while interacting with a computer simulating emotional intelligence. | Manual | Human-computer interaction & emotion | Computer game | Combination | Validation: Non-vocalized self-report methods (specific emotions rated on 7-pt Likert scale) ; Explicit externally-reported methods (behavior was coded by 3 coders using the behavioral coding system [BCS] which included four macro-categories: face, gaze direction, posture and head, and vocal behavior); Implicit externally-reported methods (heart rate, ECG) |
| Bartlett et al. (2005) | Toward Automatic Recognition of Spontaneous Facial Actions | Automatic | Product and/or software development | Robot | Non-vocalized self-reported measures | Validation: Explicit externally-reported methods (subjective judgement of happiness by observers using the turn dial technique) |
| Bezerra Alves et al. (2013) | Facial Responses to Basic Tastes in Breastfeeding and Formula-Feeding Infants | Manual | Sensory modalities & emotion | Flavor and/or taste solutions | None |  |
| Bredie et al. (2014) | A comparative study on facially expressed emotions in response to basic tastes | Manual | Sensory modalities & emotion | Flavor and/or taste solutions | Explicit externally-reported measures | Validation: Explicit externally-reported methods (manual FACS coding which was performed in a previous study; also, basic emotion was rated by a panel on a 4-point category scale) |
| Brown et al. (2014) | Developing an eBook-Integrated High-Fidelity Mobile App Prototype for Promoting Child Motor Skills and Taxonomically Assessing Children's Emotional Responses Using Face and Sound Topology. | Automatic | Product and/or software development | Multiple | None | Stimuli: mobile application, e-book mobile |
| Catia et al. (2017) | Dogs and humans respond to emotionally competent stimuli by producing different facial actions | Manual | Facial behavior & emotion expression | Multiple | None | Stimuli: animal, amusement park ride, food (unidentified), gift/present (money, unidentified), physical game |
| Chapman et al. (2017) | The Face of Distaste: A Preliminary Study | Automatic | Sensory modalities & emotion | Flavor and/or taste solutions | None |  |
| Cohn et al. (2002) | Individual differences in facial expression: Stability over time, relation to self-reported emotion, and ability to inform person identification | Both | Facial behavior & emotion expression | Comedy, joke, and/or cartoon | Combination | Validation: Implicit-externally reported methods (Facial EMG); Self-reported positive/negative emotion (it isn't clear if self-report is vocalized or nonvocalized) |
| Cole et al. (1994) | Expressive control during a disappointment: Variations related to preschoolers' behavior problems. | Manual | Human development & behavior | Toy | Combination | Validation: Explicit externally-reported (vocal quality cues as coded by trained coders); Vocalized self-reported methods (post-study interview about subject's feelings of disappointment) |
| Craig et al. (2008) | Emote aloud during learning with AutoTutor: Applying the Facial Action Coding System to cognitive-affective states during learning. | Manual | Affective states during learning | Tutoring program and/or system | Vocalized self-reported measures | Validation: Vocalized self-reported methods (vocalized report of emotions) |
| D'Mello & Graesser (2010) | Multimodal semi-automated affect detection from conversational cues, gross body language, and facial features | Both | Product and/or software development | Tutoring program and/or system | Explicit externally-reported measures | Validation: Explicit externally-reported methods (gross body language/postural features) |
| Dale et al. (1991) | Effects of dyadic participation and awareness of being monitored on facial action during exposure to humor. | Manual | Behavior towards stimuli under social and/or situational context | Multiple | None | Stimuli: film/movie clip; comedy, joke, and/or cartoon |
| Dosmukhambetova & Manstead (2012) | Fear attenuated and affection augmented: Male self-presentation in a romantic context. | Manual | Behavior towards stimuli under social and/or situational context | Film and/or movie clip | Non-vocalized self-reported measures | Validation: Non-vocalized self-reported methods (emotions rated on a 9pt scale) |
| Ekman et al. (1980) | Facial signs of emotional experience. | Manual | Facial behavior & emotion expression | Film and/or movie clip | Non-vocalized self-reported measures | Validation: Non-vocalized self-reported methods (emotions rated on a 9pt scale) |
| Espinosa-Aranda et al. (2018) | Smart doll: Emotion recognition using embedded deep learning | Automatic | Product and/or software development | Toy | None | Stimuli: doll; |
| Forestell & Mannella (2012) | More than just a pretty face. The relationship between infant’s temperament, food acceptance, and mothers’ perceptions of their enjoyment of food. | Manual | Human development & behavior | Food | None | Stimuli: food (pureed green beans) |
| Frank et al. (1997) | Behavioral markers and recognizability of the smile of enjoyment | Manual | Facial behavior & emotion expression | Film and/or movie clip | Combination | Validation: Implicit externally-reported methods (EEG); Vocalized self-reported methods (vocalized report of emotion); Non-vocalized self-reported methods (dial reporting) |
| Graesser et al. (2006) | Detection of Emotions during Learning with AutoTutor | Manual | Product and/or software development | Tutoring program and/or system | Combination | Validation: Vocalized self-reported methods (vocalized report of emotions); Explicit externally-reported methods (subjective human judgement of emotion by peers) |
| Grafsgaard et al. (2011) | Modeling Confusion: Facial Expression, Task, and Discourse in Task-Oriented Tutorial Dialogue | Manual | Affective states during learning | Tutoring program and/or system | None |  |
| Grafsgaard et al. (2013) | Automatically Recognizing Facial Indicators of Frustration: A Learning-Centric Analysis | Automatic | Affective states during learning | Tutoring program and/or system | None |  |
| Grafsgaard et al. (2014) | Predicting Learning and Affect from Multimodal Data Streams in Task-Oriented Tutorial Dialogue - Semantic Scholar | Automatic | Affective states during learning | Tutoring program and/or system | Explicit externally-reported measures | Validation: Explicit externally-reported methods (gesture and posture movements) |
| Greimel et al. (2006) | Facial and affective reactions to tastes and their modulation by sadness and joy. | Manual | Sensory modalities & emotion | Multiple | Non-vocalized self-reported measures | Stimuli: basic taste formulas, films and/or movie clips  Validation: Non-vocalized self-reported methods (emotion rated on 7pt scale) |
| Griffin & Sayette (2008) | Facial reactions to smoking cues relate to ambivalence about smoking. | Manual | Human development and/or behavior | Cigarettes | None |  |
| Gunes et al. (2019) | Live human-robot interactive public demonstrations with automatic emotion and personality prediction. | Automatic | Product and/or software development | Robot | None |  |
| Gurbez & Toga (2018) | Usage Of The Facial Action Coding System To Predict Costumer Gender Profile: A Neuro Marketing Application In TURKEY | Automatic | Product and/or software development | Food | None | Stimuli: food (chocolate) |
| Hasse et al. (2015) | Short alleles, bigger smiles? The effect of 5-HTTLPR on positive emotional expressions. | Manual | Genetics & emotion expression | Comedy, joke, and/or cartoon | None |  |
| Hung et al. (2017) | Augmenting teacher-student interaction in digital learning through affective computing | Automatic | Affective states during learning | E-book and/or audiobook | None |  |
| Jakobs et al. (1999) | Social motives and emotional feelings as determinants of facial displays: The case of smiling. | Manual | Behavior towards stimuli under social and/or situational context | Film and/or movie clip | Non-vocalized self-reported measures | Validation: Non-vocalized self-reported methods (100-millimeter emotion rating scales) |
| Johnson et al. (2017) | Positive urgency and emotional reactivity: Evidence for altered responding to positive stimuli. | Manual | Human development and/or behavior | Film and/or movie clip | Combination | Validation: Non-vocalized self-reported methods (PANAS affect questionnaire); Implicit externally-reported methods (heart rate, respiratory sinus arrhythmia, skin conductance, respiratory rate) |
| Kodra et al. (2013) | From Dials to Facial Coding: Automated Detection of Spontaneous Facial Expressions for Media Research | Both | Product and/or software development | Film and/or movie clip | Non-vocalized self-reported measures | Validation: Non-vocalized self-reported methods (dial data for emotion intensity) |
| Krumhuber & Manstead (2009) | Can Duchenne Smiles Be Feigned? New Evidence on Felt and False Smiles | Manual | Facial behavior & emotion expression | Comedy, joke, and/or cartoon | Non-vocalized self-reported measures | Validation: Non-vocalized self-reported methods (7-point emotion intensity scales) |
| Lynch (2010) | It's funny because we think it's true: Laughter is augmented by implicit preferences. | Manual | Human development & behavior | Comedy, joke, and/or cartoon | None |  |
| Lynch & Trivers (2012) | Self-deception inhibits laughter. | Manual | Behavior towards stimuli under social and/or situational context | Comedy, joke, and/or cartoon | None |  |
| Martin et al. (2013) | Towards an Affective Self-Service Agent | Manual | Product and/or software development | Self-service checkout | None |  |
| Menne et al. (2016) | Facing Emotional Reactions Towards a Robot - An Experimental Study Using FACS | Manual | Human-computer interaction & emotion | Film and/or movie clip | Non-vocalized self-reported measures | Validation: Non-vocalized self-reported methods (PANAS questionnaire) |
| Mui et al. (2017) | Children’s nonverbal displays of winning and losing: Effects of social and cultural contexts on smiles. | Manual | Behavior towards stimuli under social and/or situational context | Computer game | None |  |
| Namba et al. (2017) | Spontaneous Facial Actions Map onto Emotional Experiences in a Non-social Context: Toward a Component-Based Approach | Manual | Facial behavior & emotion expression | Film and/or movie clip | Non-vocalized self-reported measures | Validation: Non-vocalized self-reported methods (9-point emotion intensity scale) |
| Rosenstein & Oster (1988) | Differential facial responses to four basic tastes in newborns. | Manual | Sensory modalities & emotion | Flavor and/or taste solutions | None |  |
| Rossi (2013) | Emotional sophistication: Studies of facial expressions in games. | Automatic | Behavior towards stimuli under social and/or situational context | Computer game | None |  |
| Ruch (1997a) | Will the Real Relationship Between Facial Expression and Affective Experience Please Stand Up? The Case of Exhilaration. | Manual | Facial behavior & emotion expression | Comedy, joke, and/or cartoon | None |  |
| Ruch (1997b) | Extraversion, alcohol, and enjoyment | Manual | Behavior towards stimuli under social and/or situational context | Comedy, joke, and/or cartoon | None |  |
| Sayers & Sayette (2013) | Suppression on your own terms: internally generated displays of craving suppression predict rebound effects. | Manual | Human development and/or behavior | Cigarettes | None |  |
| Sayette & Hufford (1995) | Urge and affect: A facial coding analysis of smokers. | Manual | Human development and/or behavior | Cigarettes | None |  |
| Sayette & Parrott (1999) | Effects of olfactory stimuli on urge reduction in smokers. | Manual | Human development and/or behavior | Multiple | None | Stimuli: odors (n=8), cigarettes |
| Sayette et al. (2001) | A psychometric evaluation of the facial action coding system for assessing spontaneous expression. | Manual | Reliability of FACS | Multiple | None | Stimuli: odors (n=8), cigarettes |
| Sayette et al. (2005) | The effects of alcohol on cigarette craving in heavy smokers and tobacco chippers | Manual | Behavior towards stimuli under social and/or situational context | Cigarettes | None |  |
| Sayette et al. (2019) | The effects of alcohol on positive emotion during a comedy routine: A facial coding analysis. | Manual | Behavior towards stimuli under social and/or situational context | Comedy, joke, and/or cartoon | Non-vocalized self-reported measures | Stimuli: comedian Jerry Seinfeld’s stand-up act  Validation: Non-vocalized self-reported methods (mood questionnaire) |
| Schneider & Josephs (1991) | The expressive and communicative functions of preschool children's smiles in an achievement-situation. | Manual | Behavior towards stimuli under social and/or situational context | Physical game | None |  |
| Soussignan & Schaal (1996) | Forms and social signal value of smiles associated with pleasant and unpleasant sensory experience | Manual | Facial behavior & emotion expression | Odor | Non-vocalized self-reported measures | Validation: Non-vocalized self-reported methods (participants pointed at one of 5 colored cards that was associated with a hedonic rating of pleasantness) |
| Soussignan et al. (1999) | Olfactory alliesthesia in human neonates: prandial state and stimulus familiarity modulate facial and autonomic responses to milk odors. | Manual | Human development and/or behavior | Odor | Combination | Validation: Implicit externally reported methods (respiration rate, heart rate); Explicit externally-reported methods (subjective human judgement by student on 9pt Likert scale of valence of infant facial expressions) |
| Tussyadiah & Park (2018) | Consumer Evaluation of Hotel Service Robots | Automatic | Product and/or software development | Robot | Implicit externally-reported measures | Validation: Implicit externally-reported methods (skin conductance, heart rate) |
| Unzner & Schneider (1990) | Facial reactions in preschoolers: A descriptive study. | Manual | Human development and/or behavior | Physical game | None |  |
| Weiland et al. (2010) | Gustofacial and olfactofacial responses in human adults. | Manual | Sensory modalities & emotion | Multiple | Vocalized self-reported measures | Stimuli: flavor/taste solutions, odors  Validation: Vocalized self-reported methods (vocally anchored pleasantness scale) |
| ZaccheSa et al. (2015) | Facial responses to basic tastes in the newborns of women with gestational diabetes mellitus. | Manual | Sensory modalities & emotion | Flavor and/or taste solutions | None |  |
| Zhang et al. (2016) | Multimodal Spontaneous Emotion Corpus for Human Behavior Analysis | Both | Product and/or software development | Multiple | Combination | Stimuli: odor, physical game  Validation: Implicit externally-reported methods (blood pressure, respiration rate, heart rate, electrodermal activity); Non-vocalized self-reported methods (emotions and their intensities were self-reported on 5pt Likert scales) |

**Table S.4** Typology of purpose, stimuli, and validation method categories used in this review

| **Outcome** | **Categories** | **Definition** |
| --- | --- | --- |
| Facial Action Coding System (FACS) Implementation | Automatic | Action units of the FACS were coded (i.e., determined) by a machine or software program designed to assess images and/or video clips of faces |
|  | Both | Action units of the FACS were coded (i.e., determined) by both a machine or software program designed to analyze images and/or video clips of faces as well as by a human who analyzed video clips and/or live-action faces |
|  | Manual | Action units of the FACS were coded (i.e., determined) by a human who analyzed video clips and/or live-action faces |
| Purpose of Study | Affective states during learning | Any study that sought to investigate affect (emotions, mood, feelings) with respect to its impact to an individual learning and/or being educated |
|  | Behavior towards stimuli under social/situational context | Any study that sought to investigate human behavior towards a purchasable consumer product-based stimulus where study participants engaged with stimuli within/under the influence of a specific social and/or situational experimental condition |
|  | Facial behavior & emotion expression | Any study that sought to investigate how the human face physically behaves while expressing affect (emotions, mood, feelings) |
|  | Genetics & emotion expression | Any study that sought to investigate how the human genome impacts the expression of affect (emotions, mood, feelings) |
|  | Human development & behavior | Any study that sought to investigate how human physical and/or cognitive development impacts human behavior |
|  | Human-computer interaction & emotion | Any study that sought to investigate the human affective experience (emotions, mood, feelings) as it pertains to interacting with technological devices (i.e., those that function using a computer-based operational system) (emotions, mood, feelings) |
|  | Product & software development | Any study that sought to develop novel consumer product-based stimuli and/or software (e.g., computer games, mobile phone applications, automatic facial expression analysis technology, etc.) programs |
|  | Reliability of FACS | Any study that sought to investigate the reliability of the FACS as coded automatically (i.e., by a human who analyzed video clips and/or live-action faces) and/or manually (i.e., by a machine or software program designed to assess images and/or video clips of faces) |
|  | Sensory modalities & emotion | Any study that sought to investigate the relationship between human perception of sensory modalities (i.e., sight, touch, taste, hearing, smell) and affect (emotions, mood, feelings) |
| Consumer Product-Based Stimuli | Amusement park ride | A recreational ride-based activity that people purchase a ticket to experience |
|  | Animal | A creature from the Kingdom Animalia; excludes *Homo sapiens* (i.e., humans) |
|  | Cigarettes | A narrow cylinder containing psychoactive material, usually tobacco, that is rolled into thin paper for smoking |
|  | Comedy, joke, or cartoon | A cinematic film and/or film clip, cartoon show (video recording), or stand-up act show (video recorded or live) that is defined as comedic in nature (i.e., is funny and inspires laughter & amusement) |
|  | Computer games | A type of digital game played on a personal or public computer; game is designed for entertainment purposes |
|  | E-book and/or audiobook | A text-based novel that people access and read on an electronic reading device (i.e. kindle, tablet, computer, etc.) and/or an electronic device that audibly dictates/narrates the text of the novel |
|  | Film and/or movie clip | A cinematic film and/or movie clip that is not of a defined comedic nature |
|  | Flavor and/or taste solutions | A substance that is designed to stimulate the taste and olfactory receptors that help the brain perceive flavor; though these solutions are not purchased independently by consumers, they are crucial components used in the formulas/composition of consumer product-based stimuli |
|  | Food | A substance consumed to provide nutritional support for an organism |
|  | Gift/present | An item that is given willingly to someone without payment in return; item is presented as wrapped in ornate and/or decorative packaging used to disguise the gifted-item from the gift recipient |
|  | Mobile application (app) | A type of application software designed to run on a mobile device, such as a smartphone or tablet computer, which frequently serves to provide users with similar services to those accessed on personal computers |
|  | Multiple | A combination of at least two or more consumer product-based stimuli, from separate typographical categories, were assessed by the experimental population within a study |
|  | Odor | A substance that is designed to stimulate the olfactory receptors that help the brain perceive aroma; though these solutions are not purchased independently by consumers, they are crucial components used in the formulas/composition of consumer product-based stimuli |
|  | Physical game | A type of game that is physically played using stimuli (e.g., board game, jacks, cards) for the purpose of entertainment; game is not played a personal or public computer |
|  | Robot | A machine (especially one programmable by a computer) capable of carrying out a complex series of actions automatically; can be guided by an external control device or the control may be embedded within |
|  | Self-service checkout | A semi-attended customer-activated terminal (SACAT) machine which provides a mechanism for customers to process their own purchases from a retailer; machine is purchased by retailers and consumers usually have the option of using this machine during their purchase/checkout experience over a traditional human cashier-staffed checkout |
|  | Toy | An item used or especially designed for play; not a physical game or a computer game |
|  | Tutoring program and/or system | A digital software program specifically designed to aid students in learning; program may deliver content in a game-like fashion but the ultimate purpose of the software is to aid/enhance learning and not to provide entertainment |
| Validation of Affect | Combination (C) | A combination of at least two or more validation measures, from separate typographical categories, were utilized to validate the FACS determined affect (i.e. emotions) within a study |
|  | Explicit externally-reported (EER) measures | Any physical activation occurring outside of the body that can be observed/measured by another human or an instrument (AFEA, gross body movements, etc.) |
|  | Implicit externally-reported (IER) measures | Any sort of physiological activation within the body that is measured by an instrument (e.g., electrocardiograms (ECG), electroencephalograms (EEG), heart rate (HR), respiration rate, basal skin resistance, muscle tension, skin conductance, blood pressure) |
|  | Non-vocalized self-reported (NVSR) measures | Any measure where subjects did not vocalize their responses but physically respond by selecting/inputting an answer (e.g. Likert, intensity, or hedonic scales; PANAS, mood, CATA questionnaire); |
|  | None (N) | No validations measures were used to validate the FACS determined affect (i.e., emotions) within a study |
|  | Unsure if vocalized or non-vocalized (U) | Self-report measures were used to validate the FACS determined affect (i.e., emotions), but it was unclear whether the measures were reported vocally or non-vocally |
|  | Vocalized self-reported (VSR) measures | Any measure where subjects vocally communicate their affective state (i.e., emotion, mood, etc.) |

Table S.5 Definitions and interpretations of key words and frequently used terms in this systematic review

| **Term** | **Interpretation for Review** | **Definition** | **Reference** |
| --- | --- | --- | --- |
| action unit (n.) | the simplest, visible movement of a single facial muscle as coded in the Facial Action Coding System | the simplest facial movement used as a code in the Facial Action Coding System; action units are the visible signs of the operation of single facial muscles | VandenBos and American Psychological Association (2007) |
| affect (n.) | emotion or experience of emotion | any experience of feeling or emotion, ranging from suffering to elation, from the simplest to the most complex sensations of feeling, and from the most normal to the most pathological emotional reactions; Often described in terms of positive affect or negative affect, both mood and emotion are considered affective states; along with cognition and conation, affect is one of the three traditionally identified components of the mind | VandenBos and American Psychological Association (2007) |
| affect display (n.) | a form of non-verbal communication in which an emotion or affect is communicated, chiefly in humans, by a facial expression | a facial, vocal, or gestural behavior that serves as an indicator of affect | VandenBos and American Psychological Association (2007) |
| arousal (n.) | a state of physiological activation or cortical responsiveness associated with sensory stimulation and a person’s appraisal of an event or stimulus | 1. a state of physiological activation or cortical responsiveness, associated with sensory stimulation and activation of fibers from the reticular activating system  2. a state of excitement or energy expenditure linked to an emotion; usually, arousal is closely related to a person’s appraisal of the significance of an event or to the physical intensity of a stimulus | VandenBos and American Psychological Association (2007) |
| behavior (n.) | a physical manifestation or response to or towards a stimulus | 1. an organism’s activities in response to external or internal stimuli, including objectively observable activities, introspectively observable activities, and nonconscious processes  **2.** more restrictively, any action or function that can be objectively observed or measured in response to controlled stimuli; historically, behaviorists contrasted objective behavior with mental activities, which were considered subjective and thus unsuitable for scientific study | VandenBos and American Psychological Association (2007) |
| choice (n.) | referred to interchangeably as "decision" | an act of choosing between two or more possibilities | Oxford University Press (2018a) |
| choosing (v.) | to consciously decide on a course of action | to decide on a course of action; to pick out (someone or something) as being the best or most appropriate of two or more alternatives | Oxford University Press (2018b) |
| consumer (n.) | an individual that uses, consumes, or purchases products or services | an individual or an entity (e.g., a school system) that purchases (or otherwise acquires) and uses goods or services. In the context of health care, consumers are generally patients or clients who receive medical or mental health services, but they can also include parents or legal guardians responsible for obtaining services on the patients’ or clients’ behalf. | VandenBos and American Psychological Association (2007) |
| decision making (n.) | the cognitive act of processing information (implicit, explicit) that results in or underpins present or future voluntary behaviors | the cognitive process of choosing between two or more alternatives, ranging from the relatively clear cut (e.g., ordering a meal at a restaurant) to the complex (e.g., selecting a mate); psychologists have adopted two converging strategies to understand decision making: (a) statistical analysis of multiple decisions involving complex tasks and (b) experimental manipulation of simple decisions, looking at the elements that recur within these decisions | VandenBos and American Psychological Association (2007) |
| emotion (n.) | a short-term affective state that manifests implicitly and explicitly in a person; occurs subconsciously and may or may not be consciously recognized | a complex reaction pattern, involving experiential, behavioral, and physiological elements, by which an individual attempts to deal with a personally significant matter or event; the specific quality of the emotion (e.g., fear, shame) is determined by the specific significance of the event; for example, if the significance involves threat, fear is likely to be generated, if the significance involves disapproval from another, shame is likely to be generated; emotion typically involves feeling but differs from feeling in having an overt or implicit engagement with the world | VandenBos and American Psychological Association (2007) |
| facial action coding system (FACS) (n.) | a coding system for classifying facial expressions in terms of the movements of particular facial muscles | a coding system for classifying facial expressions in terms of the movements of particular facial muscles, such as the orbicularis oculi (the muscle encircling each eye) and the zygomaticus major (the muscle pulling the corners of the lips upward); elaborations of this system have been used in attempts to identify the emotional state of a person, for example, true happiness is said to be indicated by contraction of the orbicularis oculi muscle, which is not under conscious control | VandenBos and American Psychological Association (2007) |
| facial expression (n.) | movement and/or a series of movements of musculature on the face; a non-verbal communication via the face | a form of nonverbal signaling using the movement of facial muscles; an integral part of communication, facial expression also reflects an individual’s emotional state. Charles Darwin suggested that facial expressions are innate reactions that possess specific survival value, for example, a baby’s smile evokes nurturing responses in parents; although controversial, this theory has been supported by cross-cultural research, which indicates that certain facial expressions are spontaneous and universally correlated with such primary emotions as surprise, fear, anger, sadness, and happiness; display rules, however, can modify or even inhibit these expressions, as can physical conditions such as parkinsonism, which produces an expressionless, masklike countenance | VandenBos and American Psychological Association (2007) |
| feeling (n.) | a conscious recognition of emotional state | **1.** a self-contained phenomenal experience; feelings are subjective, evaluative, and independent of the sensations, thoughts, or images evoking them; feelings are inevitably evaluated as pleasant or unpleasant, but they can have more specific intrapsychic qualities, so that, for example, the affective tone of fear is experienced as different from that of anger; The core characteristic that differentiates feelings from cognitive, sensory, or perceptual intrapsychic experiences is the link of affect to appraisal; feelings differ from emotions in being purely mental, whereas emotions are designed to engage with the world  **2.** any experienced sensation, particularly a tactile or temperature sensation (e.g., pain, coldness) | VandenBos and American Psychological Association (2007) |
| judgement (n.) | the result of mental processes by which people form opinions, reach conclusions, and make critical evaluations | 1. the capacity to recognize relationships, draw conclusions from evidence, and make critical evaluations of events and people  **2.** in psychophysics, the ability to determine the presence or relative magnitude of stimuli | VandenBos and American Psychological Association (2007) |
| mood (n.) | a temporary, but relatively sustained, affective state that is characterized by valence (positive or negative) or motivation (approach or withdrawal); may or may not be consciously recognized | **1.** any short-lived emotional state, usually of low intensity (e.g., a cheerful mood, an irritable mood)  **2.** a disposition to respond emotionally in a particular way that may last for hours, days, or even weeks, perhaps at a low level and without the person knowing what prompted the state; moods differ from emotions in lacking an object, for example, the emotion of anger can be aroused by an insult, but an angry mood may arise when one does not know what one is angry about or what elicited the anger; disturbances in mood are characteristic of mood disorders | VandenBos and American Psychological Association (2007) |
| perceive (v.) | to become aware or gain factual, emotional, and contextual knowledge of something through the senses; to comprehend a stimulus | 1. to be conscious of or recognize through the senses  **2.** to understand or grasp the meaning of something | VandenBos and American Psychological Association (2007) |
| perception (n.) | a conscious comprehension of a stimulus based on emotionally- or otherwise associated information gained (implicitly, explicitly) through experiences (past, present), social and environmental contexts, etc. | the process or result of becoming aware of objects, relationships, and events by means of the senses, which includes such activities as recognizing, observing, and discriminating; these activities enable organisms to organize and interpret the stimuli received into meaningful knowledge and to act in a coordinated manner | VandenBos and American Psychological Association (2007) |
| primary emotion (n.) | anger, fear, joy (i.e. happiness), sadness, disgust, or surprise; referred to interchangeably as "universal emotions", "basic emotions", or "basic universal emotions" | any one of a limited set of emotions that typically are manifested and recognized universally across cultures. The list of primary emotions varies across different theorists; they often include fear, anger, joy, sadness, disgust, contempt, and surprise; some theorists also include shame, shyness, and guilt; also called basic emotion; core emotion | VandenBos and American Psychological Association (2007); Ekman and Friesen (1971) |
| secondary emotion (n.) | any emotion that is not a primary emotion | an emotion that is not recognized or manifested universally across cultures or that requires social experience for its construction; for some theorists, pride represents a secondary emotion, stemming from the conjunction of a primary emotion (joy) and a favorable public reaction; other secondary emotions include envy, frustration, and jealousy | VandenBos and American Psychological Association (2007) |
| purchase (v.) | to obtain or acquire something by paying for it with money | to acquire something by paying for it | Oxford University Press (2018c) |
| sensation (n.) | the subjective experience that results from excitation of sensory receptors/the senses (i.e. taste, touch, hearing, sight, smell) | **1.** the process or experience of perceiving through the senses  **2.** an irreducible unit of experience produced by stimulation of a sensory receptor and the resultant activation of a specific brain center, producing basic awareness of a sound, odor, color, shape, or taste or of temperature, pressure, pain, muscular tension, position of the body, or change in the internal organs associated with such processes as hunger, thirst, nausea, and sexual excitement; also called sense datum; sense impression; sensum | VandenBos and American Psychological Association (2007) |

**Table S.6** Action Units (AUs) investigated and/or reported on and their identified emotional interpretation by study

| **Study**  **(author, year)** | **Title** | **AUs Investigated/Reported & Their Emotion Interpretation** |
| --- | --- | --- |
| Balzarotti et al. (2014) | “I know that you know how I feel”: Behavioral and physiological signals demonstrate emotional attunement while interacting with a computer simulating emotional intelligence. | 12 actions: 6 upper face actions (AUs 1, 2, 4, 5, 6, and 7) and 6 lower face actions (AUs 9, 10, 16, 17, 18, 20); Blinks (AU 45 in the FACS system), Brow raises (AU 1+2), and Brow lower (AU 4); did not connect with emotions but in lit review they did cite that Fear = AUs 1+2+4 |
| Bartlett et al. (2005) | Toward Automatic Recognition of Spontaneous Facial Actions | None identified |
| Bezerra Alves et al. (2013) | Facial Responses to Basic Tastes in Breastfeeding and Formula-Feeding Infants | AUs 9, 10; AUs 9+10= disgust/distaste |
| Bredie et al. (2014) | A comparative study on facially expressed emotions in response to basic tastes | AUs 1, 2, 4, 7, and 14; AUs 1+2 may be associated with frustration, surprise, or anxiety; cites that AU1+AU2+AU4+AU5+AU25 = Fear and AU14 is associated with contempt and frustration |
| Brown et al. (2014) | Developing an eBook-Integrated High-Fidelity Mobile App Prototype for Promoting Child Motor Skills and Taxonomically Assessing Children's Emotional Responses Using Face and Sound Topology. | Happiness= 1+7+12+24+25+26+27; Surprise=1+5+15+25+26+27; Fear=1+4+5+15+20+24+25+26+27; Anger=4+5+15+24+25+26+27; Sadness= 1+4+7+15+24+25+26+27; Disgust=4+7+15+20+24+25 AU12, 12 + 24 = smiling; AU20 = swallowing action; AU12 + 25,26,27 = laughter; AU15 + 25,26,27 = yawning or sadness |
| Catia et al. (2017) | Dogs and humans respond to emotionally competent stimuli by producing different facial actions | AUs 1, 2, 4, 7, 14; AU4 associated with frustration (lack of AU4 associated with low engagement); AU14 associated with learning |
| Chapman et al. (2017) | The Face of Distaste: A Preliminary Study | AUs 1, 2, 4, 5, 9, 10, 12, 15, 17, 20, 26, 28, 45; AUs 4+9+10 associated with disgust, 15+12 associated with sadness/disappointment, 9+17+20+45 associated with distrust, 4+5+17= anger, 9+15+16= disgust, 1+4+5= "fear brow"/anticipation, 2+5+26= surprise |
| Cohn et al. (2002) | Individual differences in facial expression: Stability over time, relation to self-reported emotion, and ability to inform person identification | None identified |
| Cole et al. (1994) | Expressive control during a disappointment: Variations related to preschoolers' behavior problems. | AUs 1, 2, 4, 6, 12, 25, 26 |
| Craig et al. (2008) | Emote aloud during learning with AutoTutor: Applying the Facial Action Coding System to cognitive-affective states during learning. | None identified |
| D'Mello & Graesser (2010) | Multimodal semi-automated affect detection from conversational cues, gross body language, and facial features | None identified |
| Dale et al. (1991) | Effects of dyadic participation and awareness of being monitored on facial action during exposure to humor. | AUs 6, 12, 14, 15, 17, 23, 51-58 (head movements); AU 1+2+4 and AU 12+15= negative emotion |
| Dosmukhambetova & Manstead (2012) | Fear attenuated and affection augmented: Male self-presentation in a romantic context. | AUs 1, 4, 7, 12, 14, 25, 26, 43, 45, 64; AU1+2+12 with frustration, AU4+7 with confusion; AUs 7+12+25+26–45=delight |
| Ekman et al. (1980) | Facial signs of emotional experience. | AUs 2, 4, 9, 10, 12, 14; 2+4+12 indicate interest |
| Espinosa-Aranda et al. (2018) | Smart doll: Emotion recognition using embedded deep learning | AUs 1, 2, 4, 5, 6, 7, 9, 10, 11, 12, 14, 15, 16, 17, 18, 19 |
| Forestell & Mannella (2012) | More than just a pretty face. The relationship between infant’s temperament, food acceptance, and mothers’ perceptions of their enjoyment of food. | AUs 1, 2, 4, 5, 6, 7, 9, 10, 12, 14, 15, 16, 17, 18, 19, 20, 23, 24, 25 28, 30, 32, 33, 34, 37 |
| Frank et al. (1997) | Behavioral markers and recognizability of the smile of enjoyment | A1 = no distinct mouth action or sucking on the face; A2 = A1 with a negative expression on the mid-face; A3 = A1 with a negative expression on the mid-face and brows; B1 = pursing mouth; B2 is B1 with a negative expression on the mid-face; B3 = B1 with a negative expression on the mid-face and brows; C1 = mouth-gaping action; C2 = C1 with a negative expression on the mid-face; C3 = C1 with a negative expression on the mid-face and brows |
| Graesser et al. (2006) | Detection of Emotions during Learning with AutoTutor | No actual AUs from FACS were identified but the facial reactions rated were: ‘frown (AU15 or 16?)’, ‘eye widening (AU6 or 10?)’, ‘eye diminishing (AU7?)’,‘nose wrinkle (AU9)’, ‘nostril widening (AU38)’, ‘lips pressed (AU7, 8, 23, or 24?)’, ‘lips pursed (AU 18?)’, ‘lip corner up’, ‘lip corner down (AU15)', ‘mouth open (AU27?)’ and ‘tongue out (AU19 or 36?)’; Basic emotions expressed by the face were measured/rated on a four-point category scale including the categories: not at all (0), a little (1), moderate (4) and a lot (8) |
| Grafsgaard et al. (2011) | Modeling Confusion: Facial Expression, Task, and Discourse in Task-Oriented Tutorial Dialogue | AUs 5, 6, 7, 14, 17, 20, 24, 28; 5+7+20= fear, 14+17+24+28 = frustration, 6 =happiness |
| Grafsgaard et al. (2013) | Automatically Recognizing Facial Indicators of Frustration: A Learning-Centric Analysis | None identified |
| Grafsgaard et al. (2014) | Predicting Learning and Affect from Multimodal Data Streams in Task-Oriented Tutorial Dialogue - Semantic Scholar | AUs 1, 2, 4, 7, 12, 14, 43; 1+2+14 = frustration, 4+7+12 = confusion, 43 = boredom |
| Greimel et al. (2006) | Facial and affective reactions to tastes and their modulation by sadness and joy. | No actual AUs from FACS were identified but the facial reactions rated were: full-faced smiles (AUs 6+12), lip smiles (AU12), sad eyes and mouth (AUs 64 [eyes down] +15 and/or 16) |
| Griffin & Sayette (2008) | Facial reactions to smoking cues relate to ambivalence about smoking. | AUs 1, 4, 5, 12; 1+4+5= fear, 12 = affection & amusement, 4= frowning |
| Gunes et al. (2019) | Live human-robot interactive public demonstrations with automatic emotion and personality prediction. | AUs 9, 10, 12; 12= smile/happiness, 9+10= disgust |
| Gurbez & Toga (2018) | Usage Of The Facial Action Coding System To Predict Costumer Gender Profile: A Neuro Marketing Application In TURKEY | AUs 1, 4, 6, 7, 9, 10, 26, 27 = associated with distaste |
| Hasse et al. (2015) | Short alleles, bigger smiles? The effect of 5-HTTLPR on positive emotional expressions. | AUs 6, 16 = Duchenne Smile |
| Hung et al. (2017) | Augmenting teacher-student interaction in digital learning through affective computing | None identified; A list of the affective states and definitions was provided for the learners, peers, and two trained judges including: boredom, confusion, flow, frustration, delight, neutral and surprise, the emotions that were most frequently experienced in a previous study of AutoTutor; |
| Jakobs et al. (1999) | Social motives and emotional feelings as determinants of facial displays: The case of smiling. | AU4 = strongly correlated with confusion |
| Johnson et al. (2017) | Positive urgency and emotional reactivity: Evidence for altered responding to positive stimuli. | AUs 4, 6, 10, 12, 14, 15, 17, 23, 24, 26, 28, 37; 6+12= Duchenne smile, 4+10+12+26 elicited by bitter, 28 elicited by sweet, 14 elicited by bitter-sweet |
| Kodra et al. (2013) | From Dials to Facial Coding: Automated Detection of Spontaneous Facial Expressions for Media Research | AU configurations were coded as positive: 12 and 6 + 12, both of which could be accompanied by 1 + 2, 25, or 26 Negative AUs were defined by the presence of at least one of the following AUs: 9, 10, unilateral 14, 15, 20, and 1 + 4 |
| Krumhuber & Manstead (2009) | Can Duchenne Smiles Be Feigned? New Evidence on Felt and False Smiles | AUs 4, 6, 7, 12, 15, 24, 25, 26; 6+7+12+25+26+vocalization=laughter |
| Lynch (2010) | It's funny because we think it's true: Laughter is augmented by implicit preferences. | AUs 6, 12 |
| Lynch & Trivers (2012) | Self-deception inhibits laughter. | AUs 1, 2, 4, 5, 6, 7, 9, 10, 12, 13, 14, 15, 20, 24, 25; 6+12= Duchenne smile, 12= non-genuine smile, 12+6(+7 +25)= non-genuine positive behavior, 4=negative behavior, 1+2+5=surprise, 4+5= anger, 1+4=sadness |
| Martin et al. (2013) | Towards an Affective Self-Service Agent | AUs 6, 12 |
| Menne et al. (2016) | Facing Emotional Reactions Towards a Robot - An Experimental Study Using FACS | AU 6, 7, 12, 14 all associated with laughter |
| Mui et al. (2017) | Children’s nonverbal displays of winning and losing: Effects of social and cultural contexts on smiles. | AU 6, 7, 12, 14 all associated with laughter |
| Namba et al. (2017) | Spontaneous Facial Actions Map onto Emotional Experiences in a Non-social Context: Toward a Component-Based Approach | AUs 1, 2, 3, 4, 5, 6, 7, 8, 9, 10, 11, 12, 13, 14, 15, 16, 17, 18, 19, 20, 21, 22, 23 , 24, 25, 26, 27, 28, 29, 30; 23, 23+24= anger, 1 linked to sadness, surprise, and fear |
| Rosenstein & Oster (1988) | Differential facial responses to four basic tastes in newborns. | AUs 9, 10, 12, 15, 25, 38, 39; 12 + 25 + 38= intrinsic pleasantness/joy, 9, 10, 15, 39 correlated with unpleasant feelings/fear/disgust |
| Rossi (2013) | Emotional sophistication: Studies of facial expressions in games. | AUs 6, 12; 6+12= Duchenne smile |
| Ruch (1997a) | Will the Real Relationship Between Facial Expression and Affective Experience Please Stand Up? The Case of Exhilaration. | AUs 1, 2, 4, 5, 6, 7, 10, 12, 14, 23, 25, 26; 1= surprise, 7= relief, happiness, contempt, 10= disgust, 12=amusement, 10+12= contentment & disgust, 7+10+12= contempt, 5=fear, 5+10= tension, 23= sadness |
| Ruch (1997b) | Extraversion, alcohol, and enjoyment | AUs 1, 2, 4, 6, 9, 10, 18, 26, 27; 1 and/or 2), 4, 6, 9, 10 and combinations of two or more of these actions = non-sweet, 18 and  26 or 27 are taste-specific for disgust, 12=sweet, 15=bitter |
| Sayers & Sayette (2013) | Suppression on your own terms: internally generated displays of craving suppression predict rebound effects. | AUs 6, 7, 12, 13, 14; 6+7+12 =smiling/laughter; 13 and/or 14+12=humor |
| Sayette & Hufford (1995) | Urge and affect: A facial coding analysis of smokers. | AUs 2, 6, 12, 13, 14; 2+6+12= enjoyment smile, 6+12+vocalization=laughter, 12 –6+ 13, 14= non-enjoyment display |
| Sayette & Parrott (1999) | Effects of olfactory stimuli on urge reduction in smokers. | AU 14, 23, 24, 28; AU 23, 24, 28, or AU 14 expressed by itself or in combination with any other AU represented an attempt at suppression |
| Sayette et al. (2001) | A psychometric evaluation of the facial action coding system for assessing spontaneous expression. | AUs 1, 2, 6, 9, 10, 12, 14, 15, 25, 26;  AU configurations coded as positive: 12 and 6 + 12, both of which could be accompanied by 1 + 2, 25, or 26  Negative emotional expressions were defined by the absence of AU 12 and the presence of at least one of the following AUs: 9, 10, unilateral 14, 15, 20, and 1 + 4 |
| Sayette et al. (2005) | The effects of alcohol on cigarette craving in heavy smokers and tobacco chippers | AUs 1, 2, 6, 9, 10, 12, 14, 15, 25, 26;  AU configurations coded as positive: 12 and 6 + 12, both of which could be accompanied by 1 + 2, 25, or 26  Negative emotional expressions were defined by the absence of AU 12 and the presence of at least one of the following AUs: 9, 10, unilateral 14, 15, 20, and 1 + 4 |
| Sayette et al. (2019) | The effects of alcohol on positive emotion during a comedy routine: A facial coding analysis. | AUs 1, 2, 4, 6, 12, 15, 17, 25; coded as positive: 12, 6 + 12, 6 + 12 + 25, 1 + 2 + 6 + 12, coded as negative: 4, 1 + 4, 4 + 17, 12 + 15, 14 + 15, Suppressed affect included AUs 23 and 24. AUs 12 and 4, when occurring alone, were considered to reflect positive and negative AUs only if they occurred at high intensity level |
| Schneider & Josephs (1991) | The expressive and communicative functions of preschool children's smiles in an achievement-situation. | AUs 1, 2, 6, 9, 10, 12, 14, 15, 25, 26;  AU configurations coded as positive: 12 and 6 + 12, both of which could be accompanied by 1 + 2, 25, or 26  Negative emotional expressions were defined by the absence of AU 12 and the presence of at least one of the following AUs: 9, 10, unilateral 14, 15, 20, and 1 + 4 |
| Soussignan & Schaal (1996) | Forms and social signal value of smiles associated with pleasant and unpleasant sensory experience | AUs 1, 2, 6, 12, 17, 18, 19, 20, 23, 24, 25, 26; 6+12= true smile |
| Soussignan et al. (1999) | Olfactory alliesthesia in human neonates: prandial state and stimulus familiarity modulate facial and autonomic responses to milk odors. | AUs 4, 9, 10, 14, 15, 17, 18, 20, 26, 27; 9, 10, 18, 26, 27=disgust in adults/children, 4, 14, 15, 17, 20=disgust in children/infants, 12=smiling |
| Tussyadiah & Park (2018) | Consumer Evaluation of Hotel Service Robots | AUs 4, 6, 9, 12, 14, 15, 17, 23, 25, 26, 27; 4, 9, 14, 15, 17, 23= response to unpleasant odors. 12+ 25, 26, 27, or 6=response to pleasant odors |
| Unzner & Schneider (1990) | Facial reactions in preschoolers: A descriptive study. | AUs 1, 2, 4, 5, 6, 7, 10, 12, 15, 17, 22, 23, 24, 26; Joy= 6+12, Interest= I+2+5+26, Anger= 4+7+24, Sadness= I+4+15; positive emotions= 6+12, negative emotions= 4 + 7 + 10 + 15 + 17 + 22 + 23 + 24). |
| Weiland et al. (2010) | Gustofacial and olfactofacial responses in human adults. | AUs 1, 2, 4, 6, 7, 9, 10, 12, 13, 14, 15, 16, 17, 18, 19, 20, 23, 24, 25, 26, 28, 37, 84;positive facial reactions to the sweet taste= 28, negative facial reactions to the bitter, salty, sour, and umami taste= 4, 10, 15, smiling= 6+12 |
| ZaccheSa et al. (2015) | Facial responses to basic tastes in the newborns of women with gestational diabetes mellitus. | AUs 6, 12; 6+12= Duchenne smile, If 12 - 6 then the expression was coded as a non-enjoyment “social” smile, If 12 emerged before the appearance of 6 then the expression initially was scored as a social smile before transitioning into a Duchenne smile |
| Zhang et al. (2016) | Multimodal Spontaneous Emotion Corpus for Human Behavior Analysis | A1 = no distinct mouth action or sucking on the face; A2 = A1 with a negative expression on the mid-face; A3 = A1 with a negative expression on the mid-face and brows; B1 = pursing mouth; B2 is B1 with a negative expression on the mid-face; B3 = B1 with a negative expression on the mid-face and brows; C1 = mouth-gaping action; C2 = C1 with a negative expression on the mid-face; C3 = C1 with a negative expression on the mid-face and brows |
